# Multiple Isoforms of *ANRIL* in Melanoma Cells: Structural Complexity Suggests Variations in Processing

**DOI:** 10.3390/ijms18071378

**Published:** 2017-06-27

**Authors:** Debina Sarkar, Ali Oghabian, Pasani K. Bodiyabadu, Wayne R. Joseph, Euphemia Y. Leung, Graeme J. Finlay, Bruce C. Baguley, Marjan E. Askarian-Amiri

**Affiliations:** 1 Auckland Cancer Society Research Centre, University of Auckland, Faculty of Medical and Health Sciences, University of Auckland, 85 Park Rd. Grafton, 1023 Auckland, New Zealand; d.sarkar@auckland.ac.nz (D.S.); pbod004@aucklanduni.ac.nz (P.K.B.); w.joseph@auckland.ac.nz (W.R.J.); e.leung@auckland.ac.nz (E.Y.L.); b.baguley@auckland.ac.nz (B.C.B.); 2 Department of Molecular Medicine and Pathology, Faculty of Medical and Health Sciences, University of Auckland, 85 Park Rd. Grafton, 1023 Auckland, New Zealand; 3 Institute of Biotechnology, P.O. Box 56 (Viikinkaari 5), University of Helsinki, FI-00014 Helsinki, Finland; ali.oghabian@helsinki.fi

**Keywords:** melanoma, long noncoding RNA, *ANRIL*, circular RNA, isoforms, *CDKN2A*/*B*

## Abstract

The long non-coding RNA *ANRIL*, antisense to the *CDKN2B* locus, is transcribed from a gene that encompasses multiple disease-associated polymorphisms. Despite the identification of multiple isoforms of *ANRIL*, expression of certain transcripts has been found to be tissue-specific and the characterisation of *ANRIL* transcripts remains incomplete. Several functions have been associated with *ANRIL.* In our judgement, studies on *ANRIL* functionality are premature pending a more complete appreciation of the profusion of isoforms. We found differential expression of *ANRIL* exons, which indicates that multiple isoforms exist in melanoma cells. In addition to linear isoforms, we identified circular forms of *ANRIL* (*circANRIL*). Further characterisation of *circANRIL* in two patient-derived metastatic melanoma cell lines (NZM7 and NZM37) revealed the existence of a rich assortment of circular isoforms. Moreover, in the two melanoma cell lines investigated, the complements of *circANRIL* isoforms were almost completely different. Novel exons were also discovered. We also found the family of linear *ANRIL* was enriched in the nucleus, whilst the circular isoforms were enriched in the cytoplasm and they differed markedly in stability. With respect to the variable processing of *circANRIL* species, bioinformatic analysis indicated that intronic *Arthrobacter luteus* (Alu) restriction endonuclease inverted repeats and exon skipping were not involved in selection of back-spliced exon junctions. Based on our findings, we hypothesise that “*ANRIL*” has wholly distinct dual sets of functions in melanoma. This reveals the dynamic nature of the locus and constitutes a basis for investigating the functions of *ANRIL* in melanoma.

## 1. Introduction

*ANRIL* (antisense non-coding RNA in the *INK4A* locus) is a long non-coding RNA (lncRNA) that was originally identified in familial melanoma patients with a large germline deletion in the *CDKN2A/B* (also known as *INK4B-ARF-INK4A*) gene cluster and is reported to be deregulated in several malignancies such as gastric, breast, lung and bladder cancer [1,2,3,4,5]. This locus has further been identified as a hotspot for disease-associated polymorphisms and it has been consistently associated with cardiovascular disease, several cancers, diabetes, glaucoma and other conditions [6].

The most commonly accepted function of *ANRIL* is that it mediates repression of the *CDKN2A/B* locus, by association with polycomb repressor complexes (PRC1 and PRC2) [7]. However, divergent reports have suggested both concordant and discordant expression of *CDKN2A* or *CDKN2B* and *ANRIL*, again in different tissues and cell types [1,7,8,9,10,11,12]. Additionally, repression of *CDKN2A/B* by *ANRIL* via PRC1 and PRC2 is under re-evaluation due to the promiscuous nature of binding of the PRC components to RNA molecules [13].

The existence of multiple linear and circular *ANRIL* (*circANRIL*) isoforms has been reported by several groups [9,10,14]. Recently, *circANRIL* was found to be associated with ribosome biogenesis and nucleolar stress [15]. Despite the numerous splicing variants that have been described, characterisation of the isoforms in different cell lines and tissues is still incomplete, and a more complete inventory of these should be acquired (providing data on length, exon inclusion and potential secondary structure) before functional studies are undertaken. Characterisation is crucial as some of these splice variants have been reported to be cell- or tissue-specific, suggesting that they are of physiological relevance and that they mediate a variety of effects.

In some contexts, *trans*-regulation was found to be dependent on Alu motifs, which marked the promoters of *ANRIL* target genes and which were mirrored in *ANRIL* transcripts [14]. *ANRIL* transcripts containing the Alu repeats were predicted to form a stem-loop structure, suggesting RNA-chromatin interactions as potential effector mechanisms [14].

Considering this, we set out to characterise the isoforms of *ANRIL* present in melanoma cells. We chose this cell type because of the importance of the chromosome 9p21 locus including *CDKN2A*/*B* and *ANRIL* in familial and sporadic melanoma, and the availability of a large panel of New Zealand melanoma (NZM)-derived cell lines. We chose two cell lines, one producing CDKN2A (p16) protein and one not, which were being characterised as part of a separate study, but were selected for this study to ascertain whether their complement of *ANRIL* isoforms evinced the same specificity of processing, and whether their production conformed to patterns described by others. We found that these melanoma lines expressed a profusion of both linear and *circANRIL* isoform variants. Characterisation of the large number of circular isoforms, which appeared to be largely different in the two cell lines, further revealed the true complexity of this locus. We observed variability in the localisation pattern of these isoforms with linear *ANRIL* enriched in the nucleus and *circANRIL* enriched in the cytoplasm. Additionally, we found that the processing of *circANRIL* did not fit in with the published models of circRNA biogenesis that invoke either duplex formation of inverted Alu repeats located in long introns that flank exons, or “exon skipping” [16].

## 2. Results

### 2.1. Differential Expression of ANRIL Exons in Melanoma Lines

**Figure 1:**
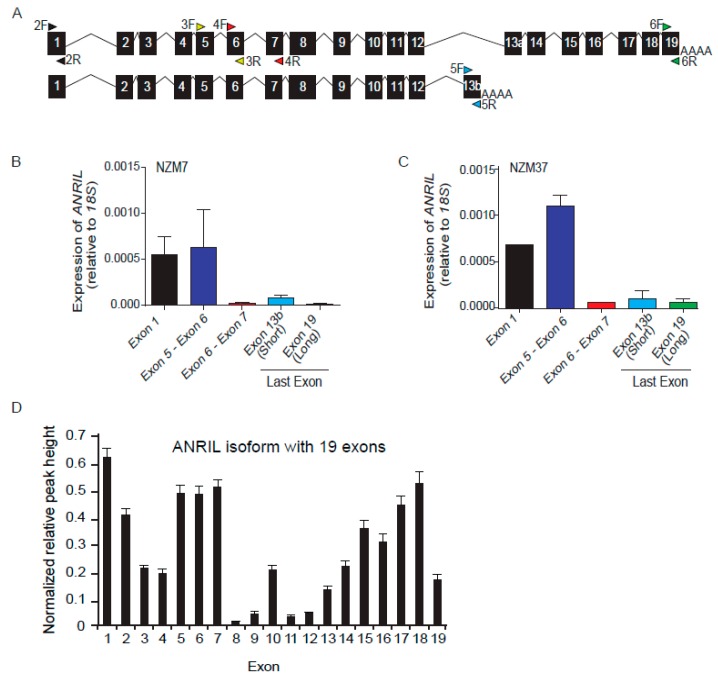
Differential expression of *ANRIL* exons/isoforms in melanoma cell lines. (**A**) Schematic of two isoforms of *ANRIL*. The arrows indicate primer sets used to detect different exons and isoforms of *ANRIL*. F and R represent forward and reverse for each primer set, respectively. Primer set 2F/2R is targeted to the first exon, and 3F/3R along with 4F/4R target middle exons which are common in all isoforms. Primer sets 5F/5R and 6F/6R target the last exons of the short (exon 13b) and long (exon 19) isoform of *ANRIL*, respectively, and were used to distinguish between them; (**B**,**C**) Differential expression of *ANRIL* exons in NZM7 and NZM37 cell lines by qPCR using primer sets described in (**A**), which indicate low expression levels for the distal exons (exons 13b and exon 19) as compared to the proximal exons (exon 1, exon 5-6); *n* = 2; (**D**) Mean expression levels of each exon of *ANRIL*, from TCGA RNA-seqV2-2013 data; *n* = 255.

To determine general expression levels, it would be ideal to consider the total length of the *ANRIL* transcript. However, due to the length of the *ANRIL* transcript, quantitative PCR (qPCR) was performed using five primer sets (Figure 1A) that covered several exons along the full length of the *ANRIL* transcript (Figure 1B,C and Figure S1A–C). Primer sets were created targeting the 5′ exon 1, the middle exons (exon 5-6 and exon 6-7) and the last 3′ exons that distinguish between the short and long isoforms (Figure 1A). To differentiate between exon 13 in the transcript with 19 exons and the last exon of the short transcript with 13 exons, we have referred to them as exon 13a and exon 13b, respectively. Differential expression of exons was observed in each of five melanoma lines which were selected from our panel at random (Figure 1B,C and Figure S1A–C). Proximal exons (exon 1 and exon 5-6) were highly expressed relative to the distal exons (exon 13b and exon 19) (Figure 1B,C and Figure S1A–C). An elevated expression of exon 13b in comparison with exon 19 (last exon of the long isoform) indicates that the shorter isoform is more abundant than the longer isoform of *ANRIL*. Higher levels of expression of exons 5-7 may reflect the contribution of these exons to shorter isoforms (Figure 1B,C and Figure S1A–C).

Differential expression of exons indicates that multiple *ANRIL* isoform variants exist in these melanoma lines. We also determined expression levels of each exon of *ANRIL* for all the skin cutaneous melanoma (SKCM) cases in TCGA database (*n* = 255; Figure 1D). This data set also revealed differential expression of all 19 exons of *ANRIL* transcripts, with exon 8 having the lowest expression (Figure 1D), emphasising the presence of multiple isoform variants in ex vivo tumour specimens.

Expression levels of exon 1 and exons 5-6 in our set of melanoma cell lines (Figure 1B,C and Figure S1A–C) were consistent with those of TCGA data set (Figure 1D). However, combined expression of exons 6-7 and distal exon 19 in the melanoma lines was low compared to the TCGA data set (Figure 1B–D and Figure S1A–C).

### 2.2. Expression and Stability of circANRIL Relative to Linear ANRIL

**Figure 2:**
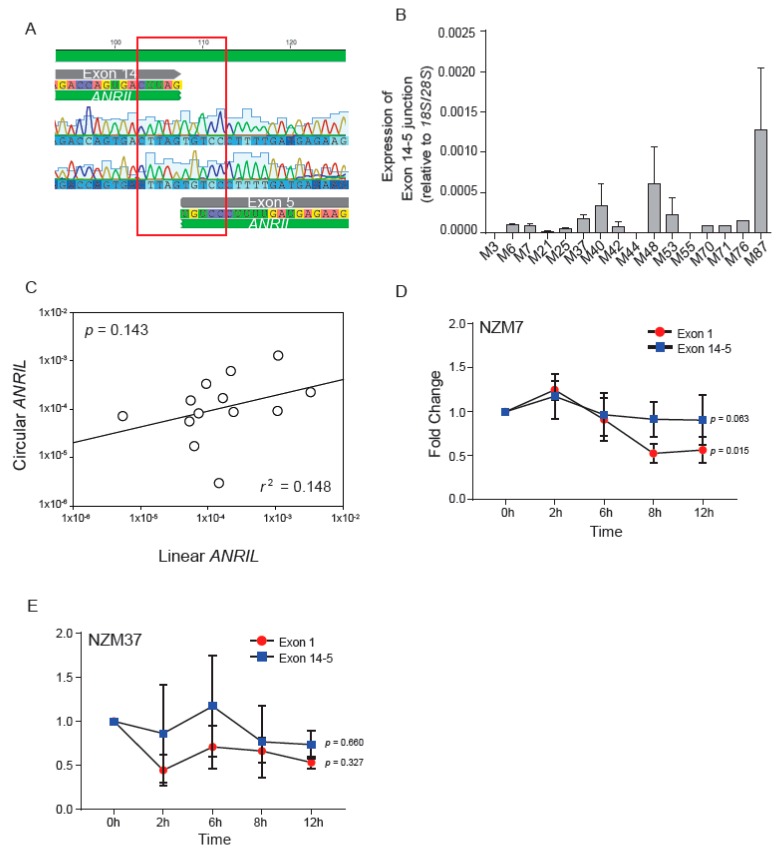
Expression and stability of *circANRIL* isoform in melanoma cell lines. (**A**) Exon 14-5 junction indicative of *circANRIL* isoform confirmed using Sanger sequencing; (**B**) Expression levels of the exon 14-5 junction indicative of *circANRIL* isoform relative to expression levels of *18S* and *28S* in 16 melanoma cell lines; (**C**) Comparison of expression levels of circular and linear *ANRIL* (*p* = 0.143, *r*^2^ = 0.148) using scatter plot and spearman correlation; (**D**,**E**) The stability of *circANRIL* represented by the exon 14-5 junction (using the in-house designed primer set) in comparison to that of linear *ANRIL* represented by expression of exon 1 after actinomycin D treatment of melanoma cells; (**D**,**E**) represent NZM7 and NZM37 cells, respectively. Expression was normalized to *GAPDH* for exon 1 and exon 14-5 junction for each time point, and fold change calculated against time 0. Linear regression analysis was done for both exon 1 and exon 14-5. Error bars indicate SEM and *n* = 3.

Burd et al. [10] reported the existence of *circANRIL* with an exon 14-5 head-to-tail junction as the predominant form in immortalized fibroblast cell lines. We set out to ascertain whether this novel isoform existed in our panel of melanoma lines. A preliminary PCR study was done using the NZM7 cell line to detect the presence of *circANRIL*. Two primer sets were used to detect the exon 14-5 junction (Figure 2A and Figure S2A). We initially used primers as reported by Burd et al. [10] and subsequently primers designed in-house to detect the same junction (Table S1). Total RNA from NZM7 cells was reverse transcribed using random hexamers, as *circANRIL* does not have a polyadenylated tail. PCR products were detected using both primer sets and sequencing of the PCR product confirmed the presence of the exon 14-5 junction, along with an exon 14-4 junction (Figure 2A and Figure S2A–D). Following this, qPCR was performed to detect expression levels of *circANRIL* in 14 melanoma cell lines using the in-house designed exon 14-5 junction primer set (Figure 2B). Only one peak was observed in the dissociation curve (Figure S2B), followed by visualization of the qPCR product on agarose gel, which further confirmed detection of only the exon 14-5 junction (Figure S2C). Except for NZM3 cells (a negative control with a deleted locus) and NZM44 and NZM55 cells, *circANRIL* was expressed in all the melanoma lines tested (Figure 2B).

Expression levels of circular (containing exon 14-5 junction) and linear *ANRIL* isoform variants for 16 samples were analyzed using SigmaPlot V13.0 (SYSTAT Software, San Jose, CA, USA). Spearman correlation was also determined for each pair of samples. No correlation was found for the expression of linear and *circANRIL* variants (Figure 2C, *p* > 0.05).

The stability of circular and linear *ANRIL* isoforms was also determined after actinomycin D treatment of melanoma cells (Figure 2D,E). Linear regression analysis was done to determine stability of *circANRIL* and linear *ANRIL*. *CircANRIL (p* = 0.063) was found to be more stable than its linear counterpart (*p* = 0.0156) in case of NZM7 (Figure 2D). Even though p value for linear exon 1 in case of NZM37 was not found to be significant, a similar trend as NZM7 was observed with *circANRIL* (*p* = 0.66) comparatively more stable than linear *ANRIL* (*p* = 0.327).

### 2.3. Characterisation of circANRIL Isoforms

Further characterization of *circANRIL* isoforms was undertaken by employing a unbiased, systematic approach using outward-facing primers against exons 2, 4, 6, 7, 8, 10, 12, 19, 14, 16, 17 and 18 (Table 1 and Figure S2E), of which primers targeting exons 4, 6 and 16 were as reported by Burd et al. [10]. Outward-facing primers could not be designed against exons 3, 5, 9, 11, 13 and 15 because these exons were of insufficient length. The priming strategy using outward-facing primers specific for exons 2, 4, 6, 7, 8, 14 and 16 identified a more comprehensive collection of *circANRIL* isoforms with various exon combinations and non-canonical, head-to-tail back-spliced junctions (Table 1 and Figure S2E).

Two melanoma cell lines, NZM7 and NZM37, were selected for *circANRIL* characterization. Transcripts from the NZM7 and NZM37 cell lines were analysed to detect and compare possible variations in the *circANRIL* isoforms present. Multiple isoforms of *circANRIL* were observed (Table 1) with various exon combinations and multiple junction variants in these two cell lines. Despite several attempts, we could not detect *circANRIL* isoforms using outward-facing primers against exons 10, 12, 17 and 18 in either cell line. However, isoforms of c*ircANRIL* using outward-facing primers against exons 8, 14 and 16 could be detected only in NZM7 and not in NZM37 cells, and the diversity of *circANRIL* species was greater for NZM7 than NZM37 cells. Interestingly, except for four *circANRIL* species (4-5-6-7-4 (7-4), 6-4-5-6 (6-4), 6-14-5-6 (14-5) and 14-4-5-6-7-14 (14-4), Table 1), the sets of circular isoforms were different in the two cell lines. Comparison of back-spliced exon junctions between NZM7 and NZM37, along with published back-spliced junctions found in the case of established cell lines by Burd et al. [10] (Figure S3A), revealed some non-canonical back-spliced junctions that were common to all the samples (exon 14-5, 7-4, 10-5 and 14-4). However, the diversity of back-spliced junctions was greater with NZM7 as compared to NZM37 cells. In addition, some novel exons were found, but only in NZM7 cells. Of all the isoforms detected in both cell lines, exons 4, 5 and 6 were most abundantly found in almost all the *circANRIL* variants. We found new exons upstream of exon 4, designated E4 (1) and E4 (2), that have not been reported previously (Figure S3B) and a novel splice variant of exon 8 (with a 1400–1705 nucleotide base region deletion, Figure S3C). All of the novel exons reported contained canonical splice sites.

### 2.4. Non-Canonical Back-Splicing of ANRIL

Published work describing circRNA in fibroblasts [16] has shown that back-spliced junctions are formed due to the presence of inverted repeat elements present in the long flanking introns. In view of the diverse *circANRIL* species and back-spliced exon junctions found in the melanoma cells, we sought to investigate conditions which facilitate the formation of back-spliced junctions of *circANRIL* in melanoma cells.

**Figure 3:**
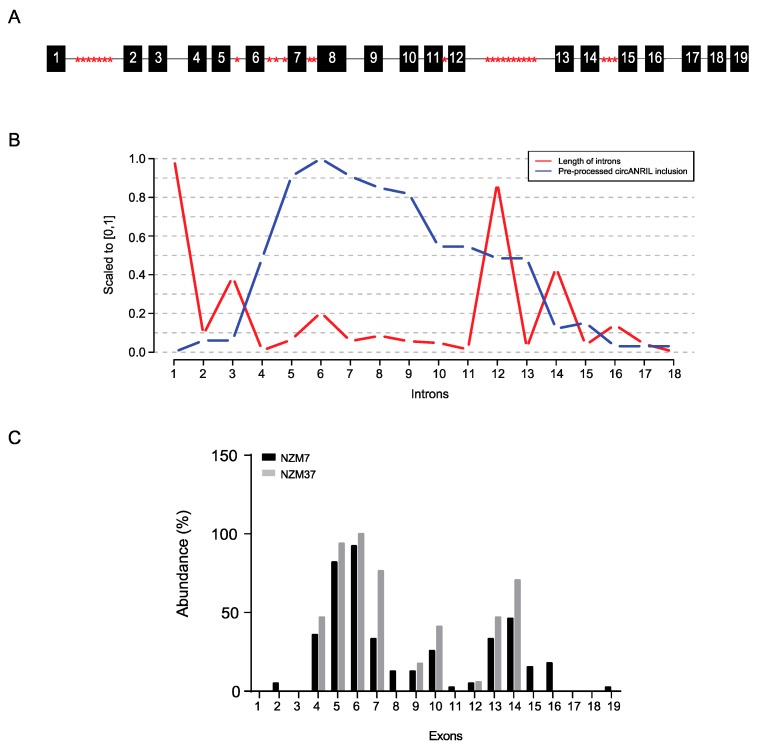
Alu repeat elements in introns and abundance of exons in *circANRIL* species in melanoma: (**A**) Schematic showing Alu elements (indicated in red) in introns of *ANRIL*; (**B**) Graph showing length of introns and the probability of introns to be included in the pre-processed *circANRIL* isoforms. The length and number of introns included in pre-processed *circANRILs* are scaled by dividing them by their maximum to unify them with the [0, 1] range; (**C**) Abundance of exons in the *circANRIL* species identified in melanoma cell lines. Relative abundance for each exon is normalised against the exon with maximum abundance for each cell line.

We examined the length of exons and introns from publicly available data for circRNA (Available on: http://circbase.org). Analysis confirmed the length of exons and introns in circular and linear RNA to be similar (Figure S4A,B), and the number of Alu elements increased with the length of the introns (Figure S4C,D). When we compared these data to *circANRIL* isoforms from both of our examined melanoma cell lines, we noted that the pairing of inverted Alu elements and the length of introns were not the only determining factors causing circularization of *ANRIL*. Alu repetitive sequences were present in introns 1, 5, 6, 7, 11, 12 and 14 of *ANRIL* (Figure 3A). Introns 5, 6, and 7 contained 1, 3, and 2 Alu elements, respectively. These introns were found to be present in pre-spliced RNA in 34, 31 and 31, respectively, of the 37 *circANRIL* isoforms found in melanoma cell lines (Figure 3B and Table 1). Among those, we found 6, 2 and 1 junctions that involved introns 5, 6 and 7, respectively. Introns 1 and 12 are the longest introns containing 7 and 10 Alu elements respectively (Figure 3A,B). The only back-spliced events including these introns were observed in *circANRIL* containing junction 10-2 and 4-12, respectively, while intron 4, which is a short intron with no Alu elements, was present in most junctions (5-7, 5-8, 5-10, 5-14, and 5-16).

The exons of *ANRIL* were also analyzed for the presence of Alu elements but as with published reports [16], Alu elements were found only in exons 7 and 12, which contain one Alu element each. These exons are not highly represented, nor do they feature frequently in *circANRIL* junctions in these cells (Figure 3C and Table 1).

Analysis was also done to predict possible back-splicing events which may be attributed to the presence of inverted Alu elements present in introns of *ANRIL* (Figure S5C). Inspection showed that several intron pairs with reverse complementary Alu repeat sequences were found for introns 14-1, 12-1, 11-1, 11-6, 11-7, 6-1, 6-7, 7-5 and 5-1, which could potentially lead to back-splicing events between exons 14-2, 12-2, 11-2, 11-7, 11-8, 6-2, 7, 7-6 and 5-2, respectively (Figure S5C and Table S2). The validated back-spliced junctions found in *circANRIL* species in the NZM cell lines (Table 1) confirmed only exon 6-2 and 7-6 back-spliced junctions. Those junctions could be formed due to presence of inverted repeat sequences present in intron pairs 6-1 and 7-1 respectively (Table S3).

The possibility of an “exon skipping” mechanism that causes lariat formation leading to formation of circRNAs has been proposed. Here, we examined this possibility for *circANRIL*. A series of exons from *ANRIL* were extracted that were perhaps skipped based on “skipped” exon events identified in RefSeq database (Table 2). However, none of these completely matched *circANRIL* variants found in this study (Table 1), therefore we excluded the involvement of an exon skipping mechanism in *circANRIL* formation.

### 2.5. Subcellular Localisation of Linear and Circular Isoforms of ANRIL Transcript 

**Figure 4:**
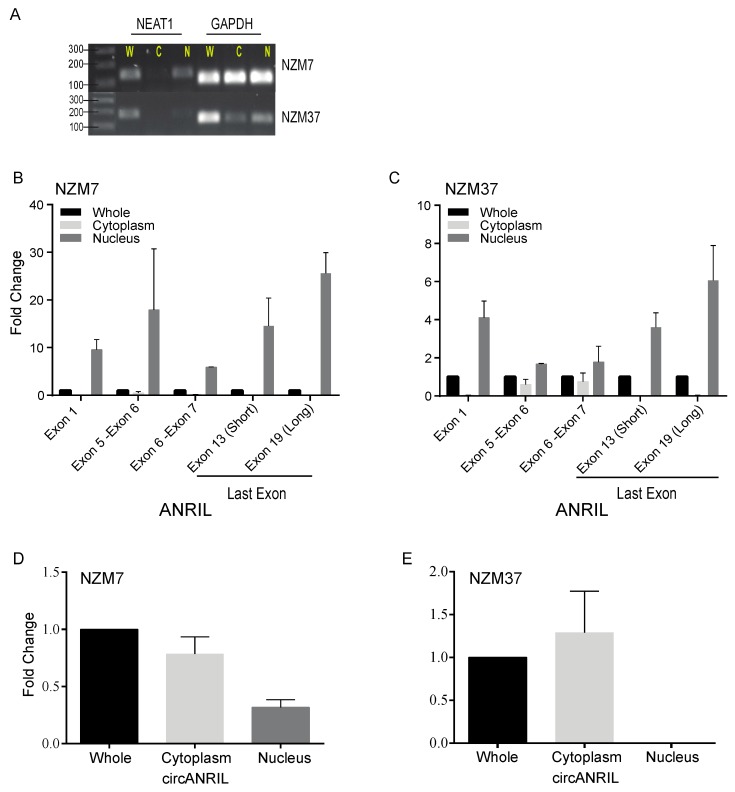
Sub-cellular localisation of circular and linear *ANRIL* transcripts in melanoma cell lines: (**A**) The purity of the cell fractions was checked by reverse transcription PCR (RT-PCR) using *NEAT1* (nuclear marker) and *GAPDH* in NZM7 and NZM37 cell extracts fractionated into nuclear and cytoplasmic fractions; (**B**,**C**) Linear *ANRIL* exons localised in the nucleus for NZM7 (**B**) and NZM37 cells (**C**), as detected by qPCR and the same primer sets as described previously (Figure 1A). The fold change relative to whole cell extracts is shown; (**D**,**E**) Localisation of *circANRIL* in fractionated cells using the exon 14-5 junction primer set. The fold change relative to whole cells is shown in NZM7 (**D**) and NZM37 (**E**) cells. Expression levels for all the targets in cytoplasmic and nuclear fractions were normalized to the relevant housekeeping gene and compared to expression levels in whole (unfractionated) cells. Error bars represent SEM (*n* = 2).

We sought to ascertain whether the diversity of linear and circ*ANRIL* isoforms was reflected in patterns of subcellular localisation. Cell extracts were separated into nuclear and cytoplasmic fractions. *NEAT1*, a nuclear RNA, and *GAPDH* were used as control transcripts to monitor the purity of the fractions (Figure 4A and Figure S6A). We performed quantitative RT-PCR for different exons of *ANRIL* (exon 1, exons 5-6, exons 6-7 and the last exons of both the short and long isoforms) in seven cell lines (NZM6, NZM7, NZM37, NZM40, NZM48, NZM 55 and NZM87) (Figure 4B,C and Figure S6B–F). The results confirmed enrichment of linear *ANRIL* in the nucleus.

Subcellular localization of *circANRIL* was also considered in the same set of melanoma lines. Quantitative RT-PCR was performed using the primer set targeted towards the exon 14-5 junction. Unlike linear *ANRIL* isoforms, most *circANRIL* transcripts localised to the cytoplasm as compared to the nucleus for almost all cell lines (Figure 4D,E and Figure S7A–E).

## 3. Discussion

We set out to characterise isoforms of *ANRIL* in melanoma cells and have shown that various exons of *ANRIL* are differentially expressed, and that multiple isoforms of *circANRIL* exist. Characterisation of *circANRIL* in these cells revealed the presence of an assortment of circular isoforms with varying lengths, exon-exon junctions and exon combinations. On further analysis, we found that formation of non-canonical back-spliced junctions was not mainly determined by Alu elements present in introns. We also observed that linear and *circANRIL* isoforms differ in their subcellular localisation, with linear isoforms enriched in the nucleus and *circANRIL* enriched in the cytoplasm.

### 3.1. Existence of Multiple Isoforms of ANRIL—Linear and Circular

Next generation sequencing (RNA-seq) has revealed differences in the abundance of *ANRIL* exons, with low expression of the central exons and high expression of the proximal and distal exons. In addition to this, of the characterised linear isoforms of *ANRIL*, transcripts with proximal exons 1-7 and distal exons 13-19 were found to be most abundant and exons from 8 to 12 were found to be skipped in the majority of mature transcripts [9,10,14].

The existence of multiple *ANRIL* species was evident through qPCR and RNA-seq data in the panel of melanoma cell lines tested here and in melanoma samples in TCGA database respectively (Figure 1B–D and Figure S1A–C). Expression levels of exons 1, 5 and 6 in our panel of melanoma cell lines (Figure 1B,C and Figure S1A–C) were consistent with that of TCGA data (Figure 1D). However, combined expression of exon 6-7 and distal exons 19 in melanoma cells was low as compared to TCGA data (Figure 1B–D and Figure S1A–C). The discrepant expression levels may reflect the homogeneity of melanoma cells as cultured lines as opposed to the heterogeneity of cell populations present in tumours (TCGA), or a cell culture as opposed to in vivo tumour environment. Genomic deletions leading to fusion of 5′-*MTAP* and 3′-*ANRIL* sequences have been described in melanomas [17]. The relatively higher abundance of 3′-*ANRIL* sequences in TCGA samples might reflect a higher frequency of melanomas with such deletions and thus divergent aetiologies.

Study of the expression levels of this locus is complex, as certain SNPs are reported to influence the expression of *ANRIL* either in *cis* or *trans* mode [11]. The diversity of transcript variants reported in both diseased and normal tissues [9,10,14], along with our data, requires that the effects of these SNPs on transcript generation should be investigated.

Burd et al. [10] first reported the existence of a circ*ANRIL* isoform. Since our initial results indicated the presence of multiple isoforms in melanoma, we investigated whether *circANRIL* is present in our melanoma cells. *CircANRIL* was found to be present in most melanoma cell lines (Figure 2B). However, the expression levels of transcripts with the exon 14-5 junction varied between cell lines (Figure 2B). The circular isoform also seemed to be more abundantly expressed than the linear isoforms. This could be due to the more stable nature of *circANRIL* (Figure 2D,E) as it is not subject to the action of exonucleases due to the absence of free ends [18]. The expression levels of linear and *circANRIL* isoforms were not significantly correlated (Figure 2C) indicating that the production of *circANRIL* and linear *ANRIL* transcripts are independent of one another. This suggests the hypothesis that linear and circular conformations are generated by independently regulated processing pathways.

### 3.2. Diversity of CircANRIL Species in Melanoma

We characterised *circANRIL* isoforms in two melanoma cell lines, NZM7 and NZM37, based on their comparable expression levels of exon 14-5 junction-containing *circANRIL* species (Figure 2B and Table 1). A wide diversity of *circANRIL* isoforms with different exon combinations, and indeed novel exons, was identified in NZM7 and NZM37 cells. However, it is striking that these two cell lines had almost wholly non-overlapping sets of circRNA species. Except for four *circANRIL* isoforms, no other variants of the identified *circANRIL* transcripts were found to be common to both NZM7 and NZM37 cells, suggesting that the forms of *circANRIL* generated were produced in differentially regulated contexts in the two cell lines (Table 1) and that they represent functional moieties rather than merely transcriptional noise.

As with previous reports [14], we identified exons 4, 5 and 6 as the most frequently included exons of all the *circANRIL* transcripts. Exons 10, 13 and 14 on the other hand were found less frequently. *CircANRIL* isoforms containing exon 8 were found only in NZM7 and not in NZM37 cells. However, full-length exon 8 was not identified in *circANRIL* (Figure S3C), but only a truncated form possessing a 1400–1705 base region deletion, suggesting that longer exons are excluded from multi-exon circular RNAs as shown (Figure 3C and Figure S5B).

Next-generation RNA-seq of HeLa cell transcriptomes revealed that the middle exons 4-12 of *linANRIL* with 19 exons were least expressed compared to the proximal and distal exons [10]. However, analysis of TCGA data for the SKCM cases revealed the expression of certain middle exons (exon numbers 5, 6, 7, 10, 13, and 14) to be quite abundant. Other exons (8, 9 11 and 12) were poorly represented. These findings are broadly consistent with the expression of the middle exons of *ANRIL* in the panel of melanoma cell lines tested here (Figure 1B–D). This indicates tissue specific expression of *ANRIL* and possible variation in isoform expression. In addition, the middle exons are found to be abundant in *circANRIL* species identified in NZM7 and NZM37 cells. The diversity of *circANRIL* transcripts identified might be taken to suggest that *circANRIL* is a mere by-product of an alternative splicing mechanism via “exon skipping” [16,19]. However, the phenomenon of exon skipping was analysed, and was shown not to be the cause for production of *circANRIL* species found in our melanoma cells (Table 2).

Diverse populations of *circANRIL* could also arise due to the presence of SNPs in the melanoma cell lines investigated, as SNPs associated with diseases including coronary artery disease (CAD), diabetes, and cancers are highly associated with *ANRIL* expression and splicing [11]. The presence of possible SNPs in melanoma cell lines therefore needs to be further investigated.

### 3.3. Back-Spliced Junctions in CircANRIL Species: Not Mediated by Intronic Alu Repeats 

Identification of isoforms is critical as the function of *ANRIL* may vary with the type of transcript variant expressed in each cell or tissue type, and hence secondary structure. Following this, further characterisation of various isoforms of *circANRIL* using outward primers revealed several novel isoforms, junctions, exons and splice variants of exons when compared to the previous study [10] (Table 1). We found certain exons such as exon 5 and 6 present in almost all isoforms of *circANRIL*. Previous studies have also confirmed the presence of exons 5 and 6, and junction 14-5 as the predominant exons and junction, respectively. However, the diversity of isoforms is very variable in different tissues examined so far [10,15] suggesting that *circANRIL* manifests a marked tissue specificity.

Jeck et al. proposed two models for biogenesis of circRNA [16,19]. The first model suggested non-canonical back-splicing of exons was due to genomic structure of long exons flanked by long introns harbouring inverted repeat elements. This preference to contain flanking Alu repeats was observed for both single- as well as multiple-exon circRNAs. The second model proposed that an alternative splicing mechanism “exon skipping” caused formation of circRNAs [16,19]. Therefore, further analysis for the presence of Alu repeat elements was done using published databases and *circANRIL* variants to investigate a possible role of these features in the *circANRIL* species identified in our melanoma cells.

Analysis of introns in unprocessed transcripts revealed Alu repeats to be present in introns 1, 5, 6, 7, 11, 12 and 14 (Figure 3A), of which several pairs of introns, e.g., 1-6, 5-7 1-12 etc. (Figure S5C) featured paired Alu elements, with sequences in reverse orientation. Back-splicing due to the presence of inverted repeat-containing introns could therefore be suggested only in the case of two of the isoforms identified in this study i.e., exon 2-6 and exon 6-7 (Table 1 and Table S3). We conclude that the reverse complementary sequences of intronic Alu elements did not contribute to the back-splicing events of *circANRIL*. As an example, exon 10-4 and exon 6-14 junctions do not fit the above category, and alternative mechanisms need to be investigated. These results suggest that although some of the circRNAs caused by back-splicing may be explained by presence of complementary Alu elements that are in reverse orientation to one another and are situated on the 5′ and 3′ side of the back-splicing region, many back-splicing events found here cannot be explained by these events and must be facilitated by other unknown factors. Exon skipping has also been considered as a plausible mechanism of circular RNA formation [19], but was not the case for *circANRIL*.

### 3.4. Localisation of Circular and Linear ANRIL 

LncRNAs must localise to their specific and diverse sites of action. Their location within cells is an important clue to assigning function. For instance, finding a lncRNA primarily in the nucleus near its site of transcription may suggest that it regulates transcription of a proximal gene (that is, regulation in *cis* or regulation of proximal loci in three dimensions) [20]. We attempted to correlate isoform heterogeneity with subcellular localisation, which might suggest hypotheses for potential roles [21].

**Figure 5:**
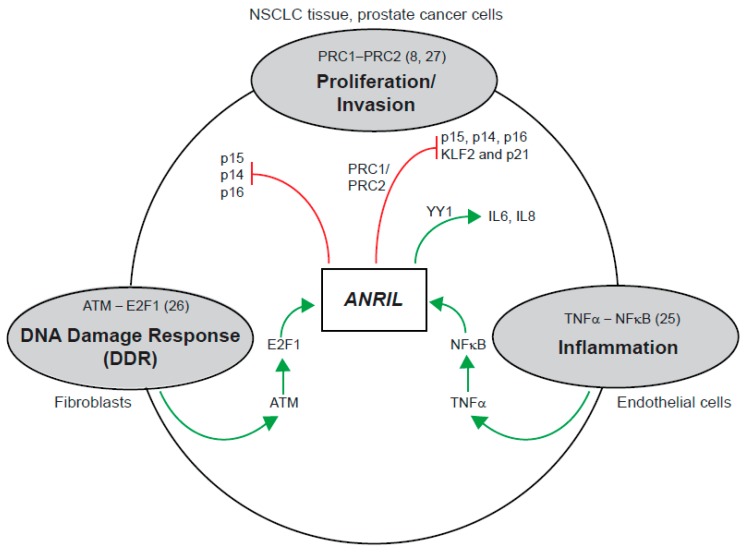
Functional overview of *ANRIL* and pathways. Schematic of pathways associated with *ANRIL*. *ANRIL* is associated with proliferation and invasion by repression of *p15*, *p16*, *p14*, *KLF2* and *p21* via PRC1 and PRC2 complexes [7,25]. *ANRIL* is induced by activation of the NF-κB pathway, and forms a complex with transcription factor *YY1* to exert transcriptional regulation on inflammatory genes *IL6* and *IL8* [23]. *ANRIL* is up-regulated by the transcription factor E2F1 in an ATM-dependent manner following DNA damage, and elevated levels of *ANRIL* suppress the expression of *CDKN2A/B* at late stages of the DNA damage response, allowing cells to return to normal at the completion of DNA repair [24]. Green lines indicate induction and red lines indicate suppression.

With respect to linear *ANRIL*, our results confirmed that both proximal and distal exons (exon 1, exon 13b and exon 19) are predominantly found in the nucleus (Figure 4B,C and Figure S6B–F). However, a small proportion of the middle exons (exon 5, exon 6 and exon 7) is present in both nucleus and cytoplasm (Figure 4B,C and Figure S6B–F). Nuclear enrichment of the linear isoforms indicates that they may regulate transcription, possibly by association with PRC2 complexes as reported previously (Figure 5) [7,8]. However, no association was found between PRC2 complexes and full length or truncated *ANRIL* in urothelial carcinoma, and also possibly in melanoma [22]. Several other functions for linear *ANRIL* have also been reported (Figure 5) [23,24], which leaves further scope for linear *ANRIL* functions to be explored in melanoma.

A major proportion of *circANRIL* was localised in the cytoplasm for most melanoma cell lines (Figure 4D,E and Figure S7A–E). This suggests its involvement in post-transcriptional regulatory mechanisms. Recently, *circANRIL* was found to disrupt exonuclease-mediated pre-rRNA processing and ribosome biogenesis in vascular smooth muscle cells and macrophages by binding to pescadillo homologue 1 (PES1), an essential 60S preribosomal assembly factor [15]. Consequently, *circANRIL* induced nucleolar stress and p53 activation resulting in induction of apoptosis and inhibition of the cell cycle, which would be expected to confer protection against the development of atheroma, and also tumour development [15]. However, cytoplasmic localisation of *circANRIL* in melanoma cell lines suggests that expression of isoforms is tissue specific, implicating *circANRIL* in alternative functions.

Additionally, based on the heterogeneity of isoforms found in our preliminary study, we propose that our cell lines possess divergent RNA processing activities. In uveal [26,27] and blue-nevus [28] melanomas, mutations in the SF3B1 splicing factor arise recurrently; in carcinomas and gliomas, the regulator *SRPK1* is overexpressed [29]. These effects have not been reported for cutaneous melanoma. It is possible that more subtle variations in splicing factor expression might underlie isoform heterogeneity in melanoma cells. For example the lncRNA *MALAT-1* has been associated with splicing [30], binds to SRSF1, at least in kidney tissue [31], and is overexpressed in metastatic melanoma [32,33]. The heterogeneity of processed lncRNAs, to which our study points, suggests the hypothesis that one class of lncRNA may regulate isoform selection of another class of lncRNA with splicing factors as intermediates. Characterisation of further lines would help clarify the processing mechanisms involved.

Systematic work to catalogue *ANRIL* isoforms in multiple cell lines of the same and different tumour types, including representatives of normal, noncancerous cells, will be needed. Future studies will combine with this study to provide a picture of how the isoforms are produced in different tumour cell lines. Only then will the function of “*ANRIL*” (or, more appropriately, the multiplicity of *ANRIL*) be disclosed.

## 4. Materials and Methods

### 4.1. Culture Conditions of Melanoma Lines

The New Zealand melanoma (NZM) cell lines were generated from surgical samples of metastatic melanoma as previously described [34]. Written consent was obtained from all patients under Auckland Area Health Board Ethics committee guidelines. NZM cell lines were grown under low oxygen conditions (5% O_2_) in order to mimic physiologically low oxygen levels in tumours. NZM cell lines were grown in α-modified minimal essential medium (αMEM; Life Technologies, Carlsbad, CA, USA) supplemented with insulin (5 µg/mL), transferrin (5 µg/mL) and sodium selenite (5 ng/mL; Roche Applied Sciences, Penzberg, Germany), 100 U/mL of penicillin, 100 µg/mL of streptomycin (PS) and 5% fetal bovine serum (FBS).

### 4.2. RNA Isolation and cDNA Synthesis

Total RNA from cultured cells or RNA from cell fractions was purified using Trizol (Life Technologies, Carlsbad, CA, USA) and treated with DNase I (Sigma-Aldrich, St. Louis, MO, USA) according to the manufacturer’s instructions. Random hexamers was used to make cDNA using 1 μg of RNA with M-MLV-Reverse Transcriptase (Sigma-Aldrich) according to manufacturer’s instructions.

### 4.3. Reverse Transcription-PCR (RT-PCR)

PCR primers (Table S1) were designed using Primer3 v. 0.4.0 software (Whitehead Institute, Cambridge, MA, USA). For non-quantitative expression analysis, PCR reactions were performed in a final volume of 20 µL, containing 1X PCR buffer, cDNA at a final 1 in 10 dilution, 0.3 μM forward and reverse primers each, 5 U Taq Polymerase, 0.2 mM deoxynucleoside triphosphates, and 2.5 mM MgCl_2_. The cycling conditions used for *NEAT1* and *GAPDH* were as follows: 95 °C for 10 min, 30 × 95 °C for 30 s, 60 °C for 45 s, and 72 °C for 1 min, and 72 °C for 15 min. Amplified products were visualized after electrophoresis on a 0.5–1% agarose gel.

### 4.4. Quantitative PCR (qPCR)

All primers (Integrated DNA Technologies, Coralville, IA, USA) used for qPCR were designed using Primer3 v. 0.4.0 software. qPCR was performed with a final 1 in 10 dilution of cDNA, 0.8 µM primers and 1x Sybr Green MasterMix (Life Technologies). Primer efficiencies were calculated using the equation: Efficiency = 10^−1/slope^. For normalisation of transcript expression levels, *18S* and *28S* ribosomal RNA (rRNA), or *GAPDH* and *HPRT* were used as reference transcripts. The average expression of these reference genes was used to calculate the relative expression of the genes of interest.

### 4.5. Gene Expression and TCGA Data Analysis

The RNAseq and exon expression data from TCGA Skin Cutaneous Melanoma (SKCM) gene expression (Illumina Hi-Seq) and exon expression (Illumina Hi-Seq) were used to examine the gene expression and exon expression respectively. Statistical analysis was performed using Sigma-Plot. Correlation analysis was performed with Pearsons Product Moment Correlation (R) and statistical significance (p). To examine the exon expression, we derived the expression level of each exon based on its chromosomal location and the average value for each exon used in bar graph (Figure 1B–D and Figure S1A–C).

### 4.6. PCR and Sequencing of the Exon 14-5 Junction of circANRIL

For qPCR and RT-PCR of *circANRIL*, primers pointing in opposite directions on exon 14-5 were designed, as described in [10], using Primer3 v. 0.4.0 software (Whitehead Institute, Cambridge, MA, USA). RT-PCR reactions were performed as mentioned before. The cycling conditions for RT-PCR used were as follows: 95 °C for 15 min, 35 × 94 °C for 30 s, 59 °C for 30 s, and 72 °C for 1 min, and 72 °C for 2 min. The correct sizes of the products were confirmed on 1% agarose gels. Excess primers in PCR products were removed using a PureLink PCR Purification Kit (Life Technology, Waltham, MA, USA) according to the manufacturer’s instructions. Concentrations of PCR products were determined using a nanodrop spectrophotometer (ND-1000, NanoDrop, ThermoFisher Scientific, Rockford, IL, USA). Sequencing was performed in two directions using ABI Prism 3730xl Genetic Analyzer with the ABI PRISM Big Dye Terminator Cycle Sequencing Ready Reaction kit, version 3.1 (Applied Biosystems, Foster City, CA, USA).

### 4.7. Stability Assay for Circular and Linear Isoforms of ANRIL

Melanoma cell lines NZM7 and NZM37 were treated with 10 µg/mL actinomycin D (Sigma-Aldrich) for 2, 6, 8 and 12 h. Total RNA from the cells was isolated for each point and cDNA synthesised as described before. Quantitative PCR was then done using primer sets for exon 1 and the exon 14-5 junction indicative of the presence of linear and circular isoforms of *ANRIL* respectively, relative to the expression of *GAPDH*. Linear regression analysis was performed to determine stability for both NZM7 and NZM37.

### 4.8. Characterisation of circANRIL Isoform Using Outward Facing Primer

Outward facing primers were used against exons 2, 4, 6, 7, 8, 14 and 16 with forward primer against the 3′ end of each exon and reverse primer against 5′ end of the same exons. RT-PCR conditions used were the same as mentioned before. When multiple bands were detected, cloning of the PCR products was conducted using a pGEM^®^-T Vector cloning kit (Promega, Madison, WI, USA). Sequencing of the resulting clones using T7 and SP6 primers was performed in two directions using ABI Prism 3730xl Genetic Analyzer as above.

### 4.9. Intron and Exon Repeat Elements Analysis

For the Alu elements enrichment analysis we extracted the DNA repetitive Alu elements from the rmsk table for human genome (*hg19*) downloaded from University of California, Santa Cruz (UCSC) genome browser. We also extracted the circRNA genomic regions from circBase [35]. We used the UCSC genes annotations in our analysis. All the analysis was performed in R [36] version 3.3.2. For the Pearson Chi-squared test and the correlation tests the relevant function in stats package of R was used. The pairwise alignment function in the Biostrings [36] package of R used to distinguish the Alu elements that are reverse compliment of one another (a minimum alignment score threshold of −200 was used). Jonckheere Terpstra test provided by the clinfun [37] R package was used to test whether there are ordered differences among values in 2 classes.

### 4.10. Exon Skipping Alternative Splicing Analysis

We extracted all the known Exon skipping from the RefSeq database using *generate Events* feature of SUPPA [37]. Next, we extracted all sets of exons of *ANRIL* that are positioned within these exon skipping events, i.e., the potential exons to form *circANRIL* isoforms. Eventually, we compared the potential list to our discovered *circANRIL* isoforms.

### 4.11. Cell Fractionation

The Lamond lab protocol was used for cell fractionation, as described in http://www.lamondlab.com/pdf/CellFractionation.pdf. Cultured melanoma cells, around 80–90% confluent, were trypsinised and pelleted. The cells were placed in hypotonic buffer A (10 mM HEPES pH 7.9, 1.5 mM MgCl_2_, 10 mM KCl, 0.5 mM DTT) for 5 min. Cells were lysed using a Wheaton Dounce tissue homogeniser (Fisher Scientific, Waltham, MA, USA). Lysed cells were then centrifuged at 1000 rpm at 4 °C for 5 min and the supernatant was collected as the cytoplasmic fraction and RNA from cytoplasmic fractions was isolated using Trizol (Life Technology) as described above. The cell pellet was applied to a sucrose cushion followed by centrifugation at 600× *g* for 15 min. The supernatant was discarded and the pellet was stored as the nuclear fraction and nuclear RNA was isolated using Trizol (Life Technology) as described above. The purity of the cell fractions was examined by RT-PCR using *NEAT1* as a nuclear marker, and the quality of RNA obtained from each fraction was assessed using *GAPDH*. qPCR was done for the cell fractions using different primer sets for linear and *circANRIL* isoforms.

## 5. Conclusions

In this study we investigated the differential expression of *ANRIL* isoforms in melanoma cell lines. The results led us to the discovery of multiple linear and circular *ANRIL* species. We also characterized the different isoform of *circANRIL* in these cell lines and revealed the presence of an assortment of circular isoforms with varying lengths, exon-exon junctions and exon combinations. By analysing the length of introns and presence of Alu elements in introns, we found that formation of non-canonical back-spliced junctions was not mainly determined by these factors. We also observed that linear and *circANRIL* isoforms differ in their subcellular localisation, with linear isoforms enriched in the nucleus and *circANRIL* enriched in the cytoplasm. Based on our findings on subcellular localization of linear and circular *ANRIL* and lack of correlation between their expression, we hypothesise that “*ANRIL*” has wholly distinct dual sets of functions in melanoma cell lines. This reveals the dynamic nature of the locus and constitutes a basis for investigating the functions of *ANRIL* in melanoma.

## Figures and Tables

**Table 1:** Isoforms of *circANRIL* identified in this study using outward-facing primers against different exons. Isoforms shown in bold indicate isoforms common to NZM7 and NZM37 cells. The back-spliced junction for each isoform is indicated in brackets beside the isoform sequence. N1 and N2 denote novel exons.

Target Exons for Outward Primers	NZM7 *CircANRIL* Isoforms	NZM37 *CircANRIL* Isoforms
Exon 2	2-5-6-2	
Exon 4	4-5-6-9-10-4 (10-4) 4-5-6-7-10-12-4 (12-4) **4-5-6-7-4 (7-4)** 4-5-6-13-14-4 (14-4) 4-5-6-10-13-14-4 (14-4) 4-5-13-14-4 (14-4) 4-5-6-12-13-14-4 (14-4) 4-5-6-13-14-4 (14-4) 4-5-6-4 (6-4) 4-5-6-10-11-12-4 (12-4)	**4-5-6-7-4 (7-4)** 4-5-6-7-13-14-4 (14-4)
Exon 6	**6-4-5-6 (6-4)** **6-14-5-6 (14-5)** 6-7-9-10-6 (10-6) 6-9-10-5-6 (10-5) 6-10-2-5-6 (10-2) 6-9-6 (9-6) 6-4(N1)-4(N2)-5-6 (6-4N1) 6-4(N2)-4-5-6 (6-4N2) 6-7-5-6 (7-5) 6-7-6 (7-6) 6-14-6 (14-6) 6-10-5-6 (10-5)	6-7-10-4-5-6 (10-4) 6-7-10-5-6 (10-5) **6-14-5-6 (14-5)** 6-7-9-10-5-6 (10-5) **6-4-5-6 (6-4)**
Exon 7	7-5-6-7 (7-5)	
Exon 8	8-5-6-8 (8-5) 8-5-6-7-8 (8-5) 8-9-10-5-6-7-8 (10-5) 8-13-14-5-6-8 (14-5) 8-10-13-14-5-6-8 (14-5)	
Exon 14	**14-4-5-6-7-14 (14-4)** 14-4-5-6-14 (14-4) 14-5-6-13N1-13-14 (14-5) 14-5-6-7-13-14 (14-5) 14-16-13N1-13-14 (16-13N1)	14-5-6-14 (14-5) 14-5-6-13-14 (14-5) **14-4-5-6-7-14 (14-4)** 14-5-6-7-13-14 (14-5) 14-4-5-6-7-9-14 (14-4) 14-4-5-6-7-13-14 (14-4) 14-5-6-7-10-13-14 (14-5) 14-5-6-7-9-10-13-14 (14-5) 14-4-5-6-7-10-13-14 (14-4) 14-5-6-7-10-12-13-14 (14-5)
Exon 16	16-15-16 (16-15) 16-5-6-7-13-14-15-16 (16-5) 16-6-7-13-14-15-16 (16-4) 14-15-16-19-5-6-10-13-14 (19-5) 16-5-6-7-13-14-15-16 (16-5)	

Outward facing primers targeted against exons 2, 4, 6, 8, 14 and 16.

**Table 2:** Exon junctions representing exon skipping events and the *circANRIL* isoforms such events may have generated.

Exon–Intron Junction	Potential *CircANRIL*
CDKN2B-AS1;SE:chr9:22029593-22032673:22032985-22046316:+	3
CDKN2B-AS1;SE:chr9:22049227-22056251:22056386-22077678:+	7, 8, 9, 10, 11, 12
CDKN2B-AS1;SE:chr9:22049227-22056251:22056386-22112319:+ CDKN2B-AS1;SE:chr9:22049227-22097257:22097363-22112319:+	7, 8, 9, 10, 11, 12, 13, 14
CDKN2B-AS1;SE:chr9:22049227-22056251:22056386-22120199:+	7, 8, 9, 10, 11, 12, 13, 14, 15, 16, 17
CDKN2B-AS1;SE:chr9:22049227-22120199:22120409-22120503:+	7, 8, 9, 10, 11, 12, 13, 14, 15, 16, 17, 18
CDKN2B-AS1;SE:chr9:22056386-22058358:22059053-22061952:+	8
CDKN2B-AS1;SE:chr9:22056386-22061952:22062025-22063943:+	8, 9
CDKN2B-AS1;SE:chr9:22056386-22063943:22064017-22077678:+	8, 9, 10, 11, 12
CDKN2B-AS1;SE:chr9:22064017-22065661:22065756-22066234:+	11
CDKN2B-AS1;SE:chr9:22064017-22066234:22066352-22077678:+	11, 12
CDKN2B-AS1;SE:chr9:22112394-22113665:22113798-22118643:+	16

## Supplementary Materials

Supplementary materials can be found at www.mdpi.com/1422-0067/18/7/1378/s1.

## References

[1] Pasmant E., Laurendeau I., Heron D., Vidaud M., Vidaud D., Bieche I. (2007). Characterization of a germ-line deletion, including the entire *INK4*/*ARF* locus, in a melanoma-neural system tumor family: Identification of *ANRIL*, an antisense noncoding RNA whose expression coclusters with *ARF*. Cancer Res..

[2] Zhang E.B., Kong R., Yin D.D., You L.H., Sun M., Han L., Xu T.P., Xia R., Yang J.S., De W. (2014). Long noncoding RNA *ANRIL* indicates a poor prognosis of gastric cancer and promotes tumor growth by epigenetically silencing of miR-99a/miR-449a. Oncotarget.

[3] Meseure D., Vacher S., Alsibai K.D., Nicolas A., Chemlali W., Caly M., Lidereau R., Pasmant E., Callens C., Bieche I. (2016). Expression of *ANRIL*-polycomb complexes-*CDKN2A*/*B*/*ARF* genes in breast tumors: Identification of a two-gene (*EZH2*/*CBX7*) signature with independent prognostic value. Mol. Cancer Res..

[4] Kang Y.H., Kim D., Jin E.J. (2015). Down-regulation of phospholipase D stimulates death of lung cancer cells involving up-regulation of the long ncRNA *ANRIL*. Anticancer Res..

[5] Zhu H., Li X., Song Y., Zhang P., Xiao Y., Xing Y. (2015). Long non-coding RNA *ANRIL* is up-regulated in bladder cancer and regulates bladder cancer cell proliferation and apoptosis through the intrinsic pathway. Biochem. Biophys. Res. Commun..

[6] Congrains A., Kamide K., Ohishi M., Rakugi H. (2013). *ANRIL*: Molecular mechanisms and implications in human health. Int. J. Mol. Sci..

[7] Yap K.L., Li S., Muñoz-Cabello A.M., Raguz S., Zeng L., Mujtaba S. (2010). Molecular interplay of the noncoding RNA *ANRIL* and methylated histone H3 lysine 27 by polycomb CBX7 in transcriptional silencing of *INK4a*. Mol. Cell.

[8] Kotake Y., Nakagawa T., Kitagawa K., Suzuki S., Liu N., Kitagawa M., Xiong Y. (2011). Long non-coding RNA *ANRIL* is required for the PRC2 recruitment to and silencing of *p15INK4B* tumor suppressor gene. Oncogene.

[9] Folkersen L., Kyriakou T., Goel A., Peden J., Malarstig A., Paulsson-Berne G., Hamsten A., Franco-Cereceda A., Gabrielsen A., Eriksson P. (2009). Relationship between CAD risk genotype in the chromosome 9p21 locus and gene expression. Identification of eight new *ANRIL* splice variants. PLoS ONE.

[10] Burd C.E., Jeck W.R., Liu Y., Sanoff H.K., Wang Z., Sharpless N.E. (2010). Expression of linear and novel circular forms of an *INK4*/*ARF*-associated non-coding RNA correlates with atherosclerosis risk. PLoS Genet..

[11] Cunnington M.S., Koref M.S., Mayosi B.M., Burn J., Keavney B. (2010). Chromosome 9p21 SNPs associated with multiple disease phenotypes correlate with *ANRIL* expression. PLoS Genet.

[12] Congrains A., Kamide K., Oguro R., Yasuda O., Miyata K., Yamamoto E., Takeya Y., Yamamoto K. (2012). Genetic variants at the 9p21 locus contribute to atherosclerosis through modulation of *ANRIL* and *CDKN2A*/*B*. Atherosclerosis.

[13] Davidovich C., Wang X., Cifuentes-Rojas C., Goodrich K.J., Gooding A.R., Lee J.T., Cech T.R. (2015). Toward a consensus on the binding specificity and promiscuity of PRC2 for RNA. Mol. Cell.

[14] Holdt L.M., Hoffmann S., Sass K., Langenberger D., Scholz M., Krohn K., Finstermeier K., Stahringer A., Wilfert W., Beutner F. (2013). Alu elements in *ANRIL* non-coding RNA at chromosome 9p21 modulate atherogenic cell functions through *trans*-regulation of gene networks. PLoS Genet..

[15] Holdt L.M., Stahringer A., Sass K., Pichler G., Kulak N.A., Wilfert W., Kohlmaier A., Herbst A., Northoff B.H., Nicolaou A. (2016). Circular non-coding RNA *ANRIL* modulates ribosomal RNA maturation and atherosclerosis in humans. Nat. Commun..

[16] Jeck W.R., Sorrentino J.A., Wang K., Slevin M.K., Burd C.E., Liu J., Marzluff W.F., Sharpless N.E. (2013). Circular RNAs are abundant, conserved, and associated with Alu repeats. RNA.

[17] Xie H., Rachakonda P.S., Heidenreich B., Nagore E., Sucker A, Hemminki K., Schadendorf D., Kumar R. (2016). Mapping of deletion breakpoints at the *CDKN2A* locus in melanoma: Detection of *MTAP*-*ANRIL* fusion transcripts. Oncotarget.

[18] Wilusz J.E. (2015). Long noncoding RNAs: Re-writing dogmas of RNA processing and stability. Biochim. Biophys. Acta.

[19] Jeck W.R., Sharpless N.E. (2014). Detecting and characterizing circular RNAs. Nat. Biotech..

[20] Cabili M.N., Dunagin M.C., McClanahan P.D., Biaesch A., Padovan-Merhar O., Regev A., Rinn J.L., Raj A. (2015). Localization and abundance analysis of human lncRNAs at single-cell and single-molecule resolution. Genome Biol..

[21] Van Heesch S., van Iterson M., Jacobi J., Boymans S., Essers P., de Bruijn E., Hao W., MacInnes A.W., Cuppen E., Simonis M. (2014). Extensive localization of long noncoding RNAs to the cytosol and mono- and polyribosomal complexes. Genome Biol..

[22] Hoffmann M., Dehn J., Droop J., Niegisch G., Niedworok C., Szarvas T., Schulz W. (2015). Truncated Isoforms of lncRNA *ANRIL* are overexpressed in bladder cancer, but do not contribute to repression of *INK4* tumor suppressors. Non-Coding RNA.

[23] Zhou X., Han X., Wittfeldt A., Sun J., Liu C., Wang X., Gan L.M., Cao H., Liang Z. (2016). Long non-coding RNA *ANRIL* regulates inflammatory responses as a novel component of NF-κB pathway. RNA Biol..

[24] Wan G., Mathur R., Hu X., Liu Y., Zhang X., Peng G. (2013). Long non-coding RNA ANRIL (CDKN2B-AS) is induced by the ATM-E2F1 signaling pathway. Cell Signal..

[25] Nie F.Q., Sun M., Yang J.S., Xie M., Xu T.P., Xia R., Liu Y.W., Liu X.H., Zhang E.B., Lu K.H. (2015). Long noncoding RNA *ANRIL* promotes non-small cell lung cancer cell proliferation and inhibits apoptosis by silencing KLF2 and P21 expression. Mol. Cancer Ther..

[26] Decatur C.L., Ong E., Garg N., Anbunathan H., Bowcock A.M., Field M.G., Harbour J.W. (2016). Driver mutations in uveal melanoma: Associations with gene expression profile and patient outcomes. JAMA Ophthalmol..

[27] Alsafadi S., Houy A., Battistella A., Popova T., Wassef M., Henry E., Tirode F., Constantinou A., Piperno-Neumann S., Roman-Roman S. (2016). Cancer-associated *SF3B1* mutations affect alternative splicing by promoting alternative branchpoint usage. Nat. Commun..

[28] Griewank K.G., Muller H., Jackett L.A., Emberger M., Moller I., van de Nes J.A.P., Zimmer L., Livingstone E., Wiesner T., Scholz S.L. (2017). *SF3B1* and *BAP1* mutations in blue nevus-like melanoma. Mod. Pathol..

[29] Bullock N., Oltean S. (2017). The many faces of SRPK1. J. Pathol..

[30] Lin R., Roychowdhury-Saha M., Black C., Watt A.T., Marcusson E.G., Freier S.M., Edgington T.S. (2011). Control of RNA processing by a large non-coding RNA over-expressed in carcinomas. FEBS Lett..

[31] Hu M., Wang R., Li X., Fan M., Lin J., Zhen J., Chen L., Lv Z. (2017). LncRNA *MALAT1* is dysregulated in diabetic nephropathy and involved in high glucose-induced podocyte injury via its interplay with β-catenin. J. Cell. Mol. Med..

[32] Sun Y., Cheng H., Wang G., Yu G., Zhang D., Wang Y., Fan W., Yang W. (2017). Deregulation of miR-183 promotes melanoma development via lncRNA *MALAT1* regulation and ITGB1 signal activation. Oncotarget.

[33] Tian Y., Zhang X., Hao Y., Fang Z., He Y. (2014). Potential roles of abnormally expressed long noncoding RNA UCA1 and Malat-1 in metastasis of melanoma. Melanoma Res..

[34] Marshall E.S., Matthews J.H., Shaw J.H., Nixon J., Tumewu P., Finlay G.J., Holdaway K.M., Baguley B.C. (1994). Radiosensitivity of new and established human melanoma cell lines: Comparison of [^3^H]thymidine incorporation and soft agar clonogenic assays. Eur. J. Cancer.

[35] Glazar P., Papavasileiou P., Rajewsky N. (2014). CircBase: A database for circular RNAs. RNA.

[36] R. Development Core Team (2011). R: A Language and Environment for Statistical Computing.

[37] Alamancos G.P., Pagès A., Trincado J.L., Eyras E. (2015). Leveraging transcript quantification for fast computation of alternative splicing profiles. RNA.

