# Correction: Sarkar, D., et al. Multiple Isoforms of *ANRIL* in Melanoma Cells: Structural Complexity Suggests Variations in Processing. *Int. J. Mol. Sci.* 2017, *18*, 1378

**DOI:** 10.3390/ijms19051343

**Published:** 2018-05-02

**Authors:** Debina Sarkar, Ali Oghabian, Pasani K. Bodiyabadu, Wayne R. Joseph, Euphemia Y. Leung, Graeme J. Finlay, Bruce C. Baguley, Marjan E. Askarian-Amiri

**Affiliations:** 1 Auckland Cancer Society Research Centre, University of Auckland, Faculty of Medical and Health Sciences, University of Auckland, 85 Park Rd. Grafton, 1023 Auckland, New Zealand; d.sarkar@auckland.ac.nz (D.S.); pbod004@aucklanduni.ac.nz (P.K.B.); w.joseph@auckland.ac.nz (W.R.J.); e.leung@auckland.ac.nz (E.Y.L.); b.baguley@auckland.ac.nz (B.C.B.); 2 Department of Molecular Medicine and Pathology, Faculty of Medical and Health Sciences, University of Auckland, 85 Park Rd. Grafton, 1023 Auckland, New Zealand; 3 Institute of Biotechnology, P.O. Box 56 (Viikinkaari 5), University of Helsinki, FI-00014 Helsinki, Finland; ali.oghabian@helsinki.fi

The authors wish to make the following corrections to this paper [1]: Some errors in the *circANRIL* isoforms were reported in Table 1, Figure S2E, and Figure S3A. The *circANRIL* isoforms have been corrected in Table 1: Under “NZM7 *CircANRIL* Isoforms” (exon 6), 6-10-4-5-6 (10-4) has been added, and 6-9-6 (9-6), 6-7-6 (7-6), and 6-14-6 (14-6) have been deleted; 6-7-9-10-6 (10-6) has been modified to 6-7-9-10-5-6 (10-5). For exon 16 in the second to last row, 14-15-16-19-5-6-10-13-14 (19-5) has been corrected to 16-19-5-6-10-13-14-15-16 (19-5), and in the last row, 16-5-6-7-13-14-15-16 (16-5) has been modified to 16-4-5-6-7-13-14-15-16 (16-4).

Accordingly, Figure S2E has been modified for exon 6, for which 4-5-6-10 has been added, and 6-9, 6-7, and 6-14 have been deleted; 6-7-9-10 has been modified to 5-6-7-9-10. Figure S2E has also been modified for exon 16, for which 5-6-7-13-14-15-16 has been modified to 4-5-6-7-13-14-15-16.

Figure S3A has also been modified, in which junctions 10-6, 9-6, 7-6, and 14-6 have been deleted in the NZM7 panel and junction 6-5 which was added to the Burd et al. [2] panel. 

These corrections induced a few minor changes in the text of the Results and Discussion sections.

In the section “2.3. Characterisation of *circANRIL* Isoforms” of Results:

On page 6, the sixth sentence of paragraph one of the original publication [1] incorrectly stated, “Interestingly, except for four *circANRIL* species (4-5-6-7-4 (7-4), 6-4-5-6 (6-4), 6-14-5-6 (14-5), and 14-4-5-6-7-14 (14-4) Table 1), the sets of circular isoforms were different in the two cell lines”. Instead, this statement should read, “Interestingly, except for five *circANRIL* species (4-5-6-7-4 (7-4), 6-4-5-6 (6-4), 6-14-5-6 (14-5), 14-4-5-6-7-14 (14-4), and 6-7-9-10-5-6 (10-5) Table 1), the sets of circular isoforms were different in the two cell lines”.

In the Section “2.4. Non-Canonical Back-Splicing of *ANRIL*” of Results:

On page 8, there are some mistakes in the third sentence and the fourth sentence in the first paragraph. This paragraph should be corrected to, “Analysis was also done to predict possible back-splicing events which may be attributed to the presence of inverted Alu elements present in introns of *ANRIL* (Figure S5C). Inspection showed that several intron pairs with reverse complementary Alu repeat sequences were found for introns 14-1, 12-1, 11-1, 11-6, 11-7, 6-1, 6-7, 7-5 and 5-1, which could potentially lead to back-splicing events between exons 14-2, 12-2, 11-2, 11-7, 11-8, 6-2, 7, 7-6 and 5-2, respectively (Figure S5C and Table S2). The validated back-spliced junctions found in *circANRIL* species in the NZM cell lines (Table 1) confirmed only the exon 6-2 back-spliced junction. This junction can be formed due to the presence of inverted repeat sequences present in intron pair 6-1 (Table S3)”.

In the Discussion section:

On page 11, Section 3.3, the third-eighth lines in paragraph three of the original publication [1] incorrectly stated, “Back-splicing due to the presence of inverted repeat-containing introns could therefore be suggested only in the case of two of the isoforms identified in this study i.e., exon 2-6 and exon 6-7 (Table 1 and Table S3). We conclude that the reverse complementary sequences of intronic Alu elements did not contribute to the back-splicing events of *circANRIL*. As an example, exon 10-4 and exon 6-14 junctions do not fit the above category, and alternative mechanisms need to be investigated”. Instead this sentence should read, “Back-splicing due to the presence of inverted repeat-containing introns could therefore be suggested only in the case of one of the isoforms identified in this study, i.e., exon 2-6 (Table 1 and Table S3). We conclude that the reverse complementary sequences of intronic Alu elements did not contribute to the back-splicing events of *circANRIL*. As an example, the exon 10-4 junction does not fit the above category, and alternative mechanisms need to be investigated”.

These changes have no material impact on the conclusions of our paper. The authors would like to apologize for any inconvenience caused to the readers by these changes.

## Figures and Tables

**Figure S2E:**
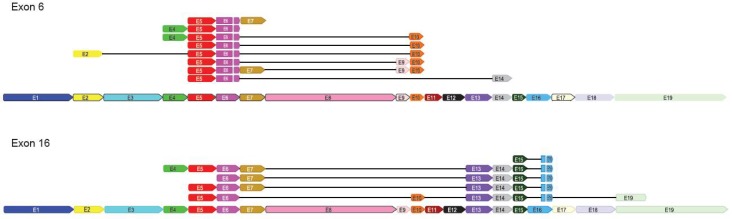
Alignment of isoforms derived from an outward-facing priming strategy against different linear *ANRIL* transcripts for exons 6 and 16.

**Figure S3A:**
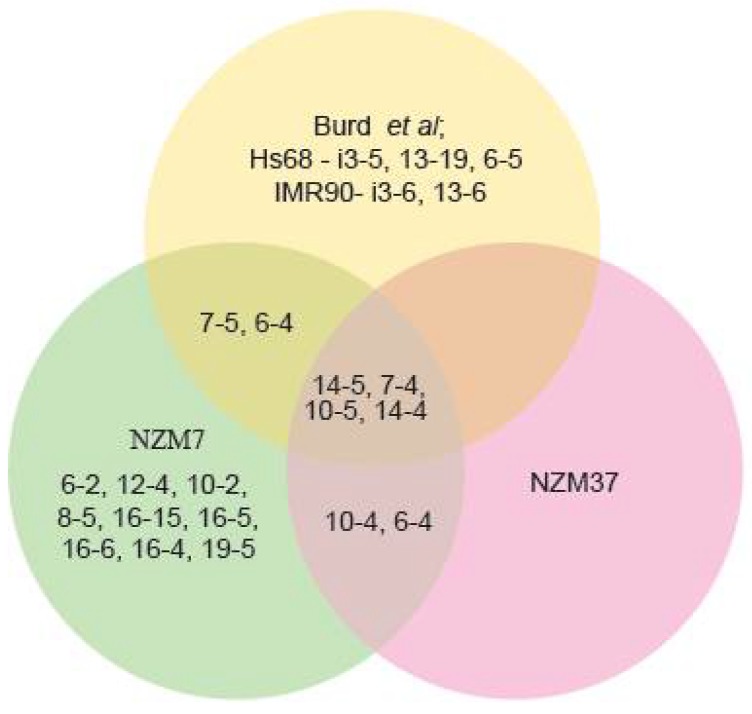
Venn diagram indicating common and novel back-spliced exon junctions in NZM7 cells, NZM37 cells, and the published dataset in Burd et al. [2].

**Table 1:** Isoforms of *circANRIL* identified in this study using outward-facing primers against different exons. Isoforms shown in bold indicate isoforms common to NZM7 and NZM37 cells. The back-spliced junction for each isoform is indicated in brackets beside the isoform sequence. N1 and N2 denote novel exons.

Target Exons for Outward Primers	NZM7 *CircANRIL* Isoforms	NZM37 *CircANRIL* Isoforms
Exon 2	2-5-6-2	
Exon 4	4-5-6-9-10-4 (10-4) 4-5-6-7-10-12-4 (12-4) **4-5-6-7-4 (7-4)** 4-5-6-13-14-4 (14-4) 4-5-6-10-13-14-4 (14-4) 4-5-13-14-4 (14-4) 4-5-6-12-13-14-4 (14-4) 4-5-6-13-14-4 (14-4) 4-5-6-4 (6-4) 4-5-6-10-11-12-4 (12-4)	**4-5-6-7-4 (7-4)** 4-5-6-7-13-14-4 (14-4)
Exon 6	**6-4-5-6 (6-4)** **6-14-5-6 (14-5)** **6-7-9-10-5-6 (10-5)** 6-9-10-5-6 (10-5) 6-10-2-5-6 (10-2) 6-4(N1)-4(N2)-5-6 (6-4N1) 6-4(N2)-4-5-6 (6-4N2) 6-7-5-6 (7-5) 6-10-5-6 (10-5) 6-10-4-5-6 (10-4)	6-7-10-4-5-6 (10-4) 6-7-10-5-6 (10-5) **6-14-5-6 (14-5)** **6-7-9-10-5-6 (10-5)** **6-4-5-6 (6-4)**
Exon 7	7-5-6-7 (7-5)	
Exon 8	8-5-6-8 (8-5) 8-5-6-7-8 (8-5) 8-9-10-5-6-7-8 (10-5) 8-13-14-5-6-8 (14-5) 8-10-13-14-5-6-8 (14-5)	
Exon 14	**14-4-5-6-7-14 (14-4)** 14-4-5-6-14 (14-4) 14-5-6-13N1-13-14 (14-5) 14-5-6-7-13-14 (14-5) 14-16-13N1-13-14 (16-13N1)	14-5-6-14 (14-5) 14-5-6-13-14 (14-5) **14-4-5-6-7-14 (14-4)** 14-5-6-7-13-14 (14-5) 14-4-5-6-7-9-14 (14-4) 14-4-5-6-7-13-14 (14-4) 14-5-6-7-10-13-14 (14-5) 14-5-6-7-9-10-13-14 (14-5) 14-4-5-6-7-10-13-14 (14-4) 14-5-6-7-10-12-13-14 (14-5)
Exon 16	16-15-16 (16-15) 16-5-6-7-13-14-15-16 (16-5) 16-6-7-13-14-15-16 (16-4) 16-19-5-6-10-13-14-15-16 (19-5) 16-4-5-6-7-13-14-15-16 (16-4)	

Outward facing primers targeted against exons 2, 4, 6, 8, 14 and 16.
