# Supplementary material for: Multiple Isoforms of ANRIL in Melanoma Cells: Structural Complexity Suggests Variations in Processing
Source: Int J Mol Sci. 2017 Jun 27;18(7):1378. doi: 10.3390/ijms18071378 (PMC5535871; doi:10.3390/ijms18071378)
Supplement: Supplementary file 1 [file ijms-18-01378-s001.zip › ijms-202411-supplymentary figures.pdf]

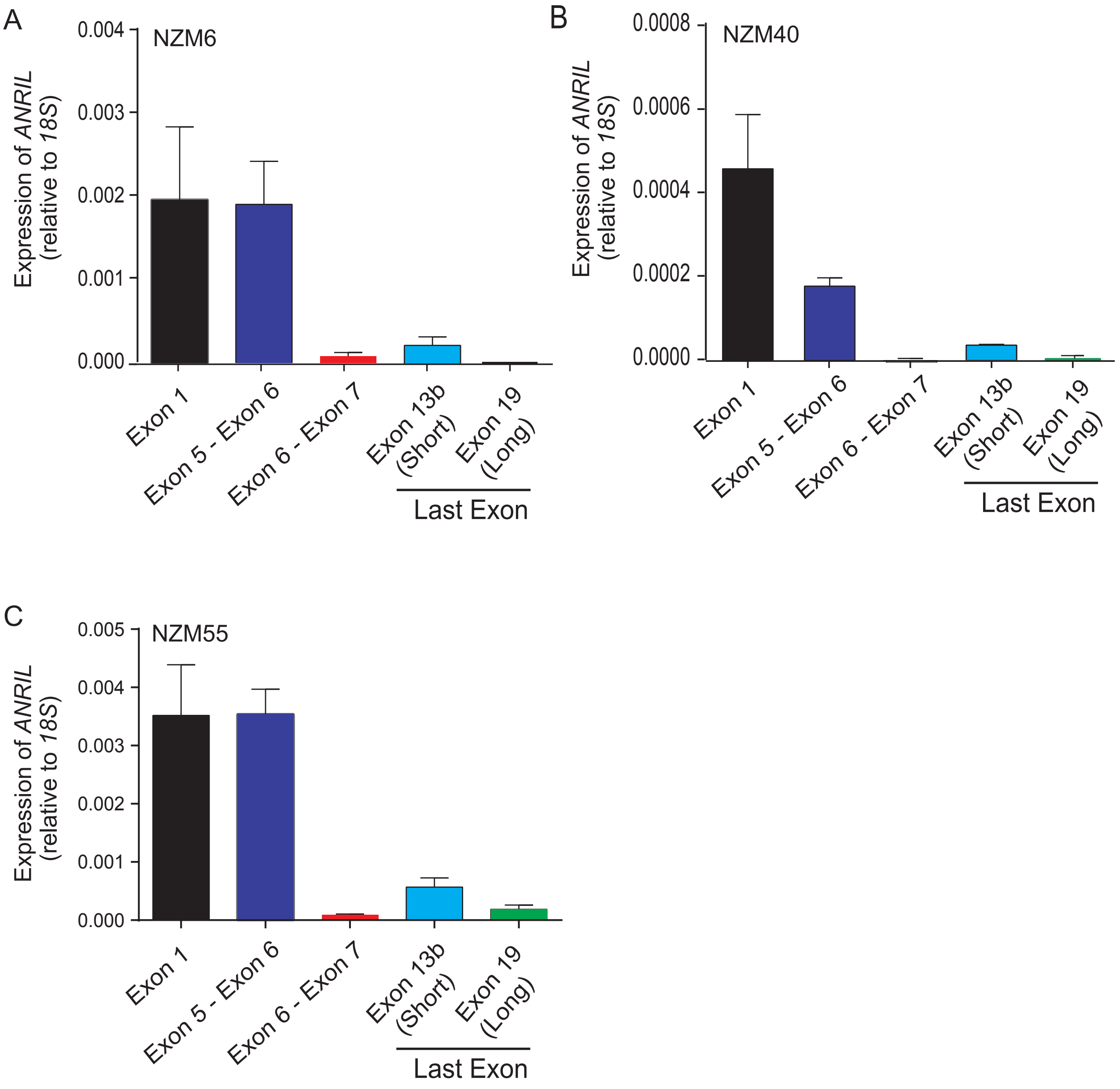

Figure S1. Differential expression of ANRIL exons/isoforms in melanoma cell lines. (A-C) Differential expression of different exons of ANRIL in NZM6 (A), NZM40 (B) and NZM55 (C) cell lines as determined by qPCR and the primer sets described in Figure 1A, which indicate low expression levels for the distal exons 13b and 19 as compared to the proximal exons 1, 5 and 6.

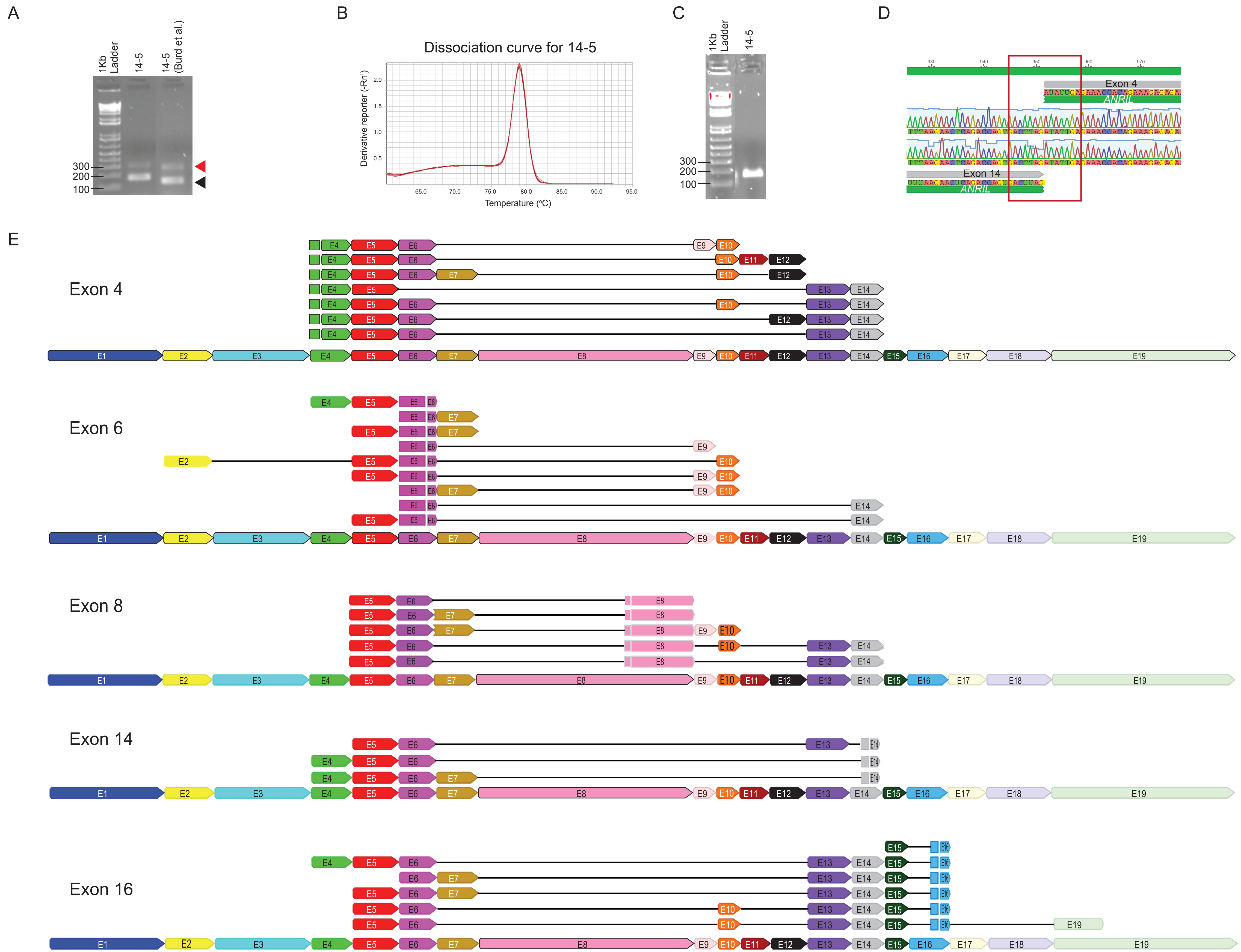

Figure S2. Identification of non-canonical backspliced 14-5 and 14-4 junctions and alignment of circANRIL isoforms with linear ANRIL transcript. (A) Detection of exon 14-5 (black arrowhead) and 14-4 (red arrowhead) junction by RT-PCR using two primer sets, one designed in-house and the other as used by Burd et al. (11) targeted against the exon 14-5 junction. (B) Dissociation curve showing one peak, thereby indicating one product obtained using the in-house designed primer set targeting the exon 14-5 junction. C) The presence of one product validated further by visualization of the same qPCR product on 1% agarose gel. (D) Exon 14–4 junction indicative of circANRIL isoform confirmed using Sanger sequencing. (E) Alignment of isoforms derived from an outward-facing priming strategy against different linear ANRIL transcripts for exons 4, 6, 8, 14 and 16.

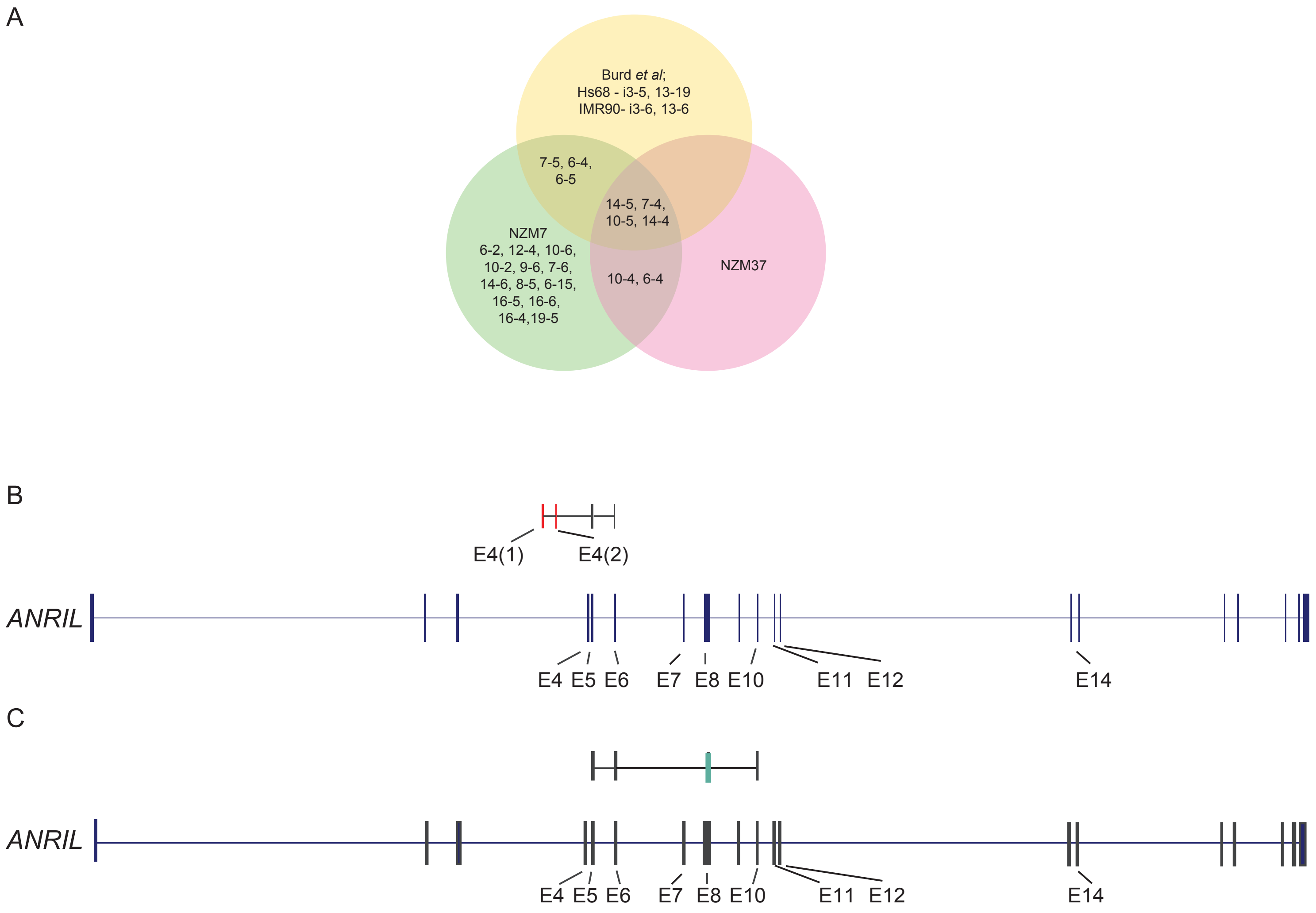

Figure S3. Back-spliced junctions found and alignment of new exons and splice variants to linear ANRIL transcript. (A) Venn diagram indicating common and novel back-spliced exon junction between NZM7, NZM37 and published dataset in Burd et al. (B) Novel exons found upstream of exon 4 have been designated as E4(1) and E4(2) indicated in red. (C) Splice variant of exon 8 indicated in blue with a deletion of 1400 – 1705 base region. Mapped using 2013 (GRCh38/hg38) version of Genome Browser. i3 indicates intron 3.

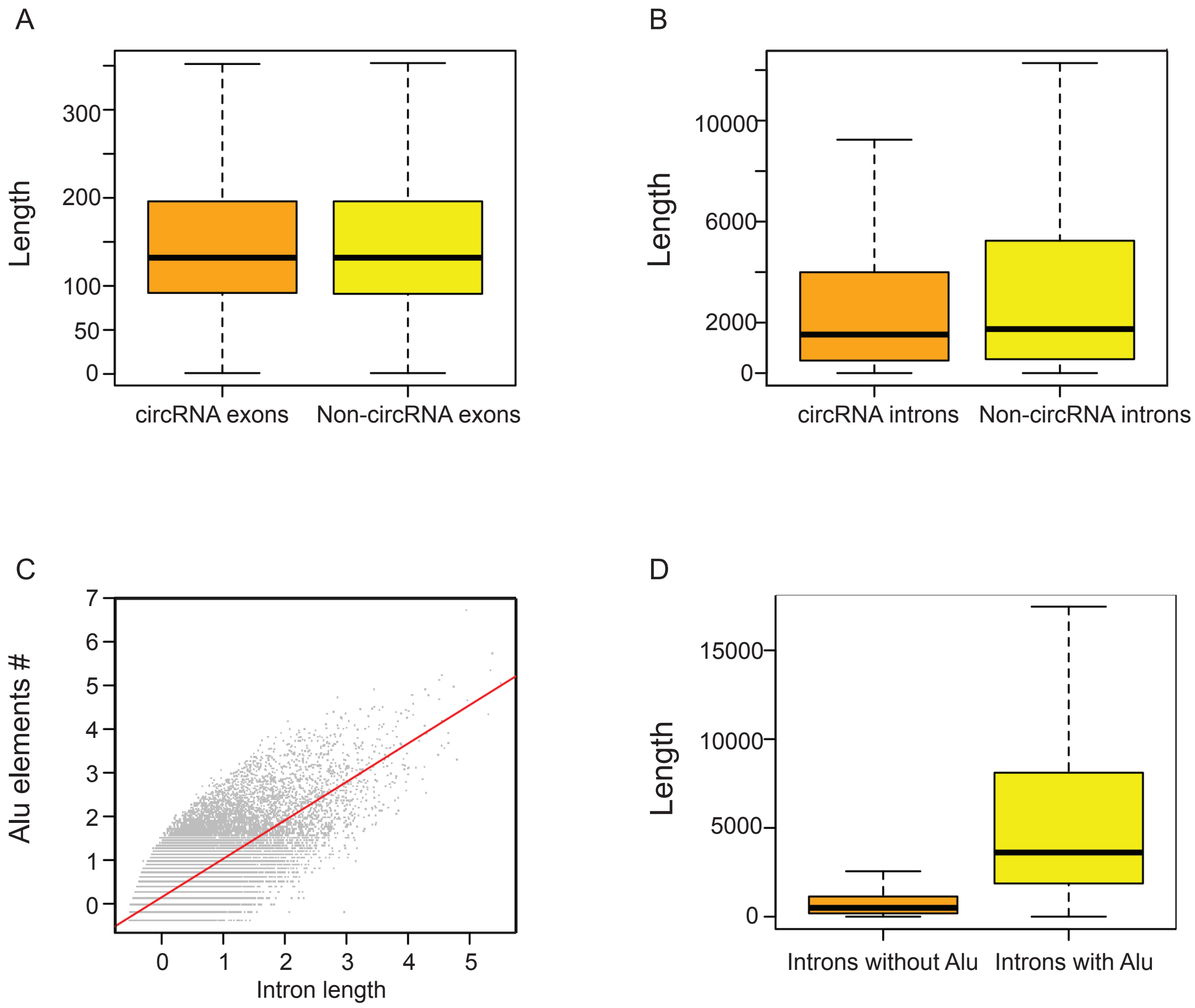

Figure S4. Introns and exons of circRNAs and non-circular RNA: comparison using circbase data. (A) Comparison of exon lengths (number of nucleotides) of circRNA and non-circRNA. (B) Comparison of intron lengths (number of nucleotides) in unprocessed or primary transcripts from which mature isoforms are derived for circRNA and non-circRNA. (C) Positive correlation of the number of Alu repeat elements and the length of introns (in kb) using Jonckheere Trend test (54, 55) ($R = 0.74$, $p = < 2.2$ e-16). (D) Comparison of the length of unspliced introns (number of nucleotides) with and without Alu elements (Pearson's Chi-squared test $p = 0.00009$). Horizontal lines indicate median and whiskers represent 1.5 times the interquartile range with outliers removed.

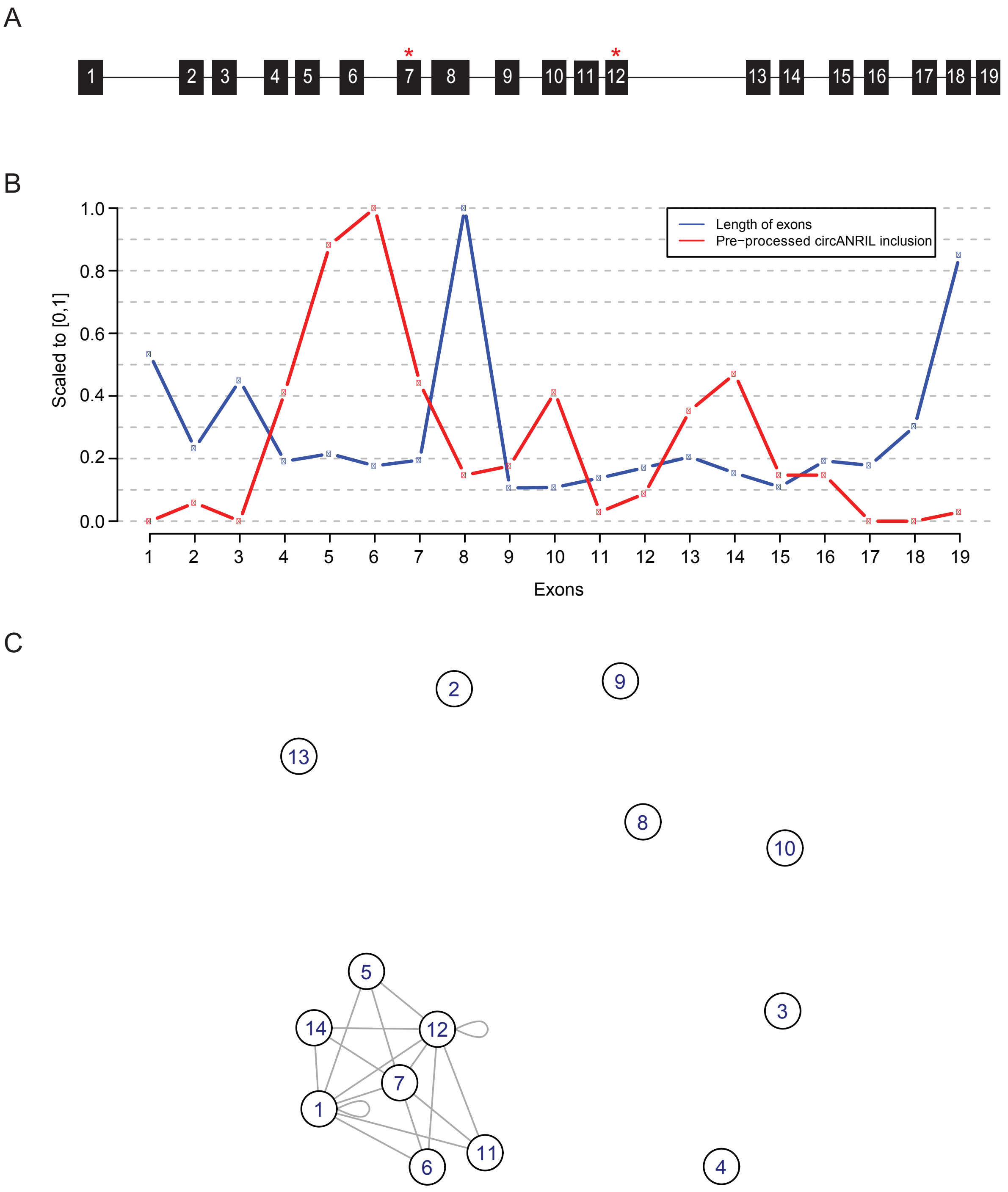

Figure S5. Analysis of Alu elements in exons of ANRIL and introns reverse complement network. (A) Schematic showing Alu elements (indicated in red) in exons of ANRIL. (B) Length of exons of ANRIL and probability of inclusion in circANRIL species. (C) Introns of ANRIL that feature at least one pair of reverse complementary Alu elements. The nodes in the graph are the introns of ANRIL and two nodes are connected via an edge if at least one pair of Alu elements exist on the two exons that are reverse complementary to one-another. Reverse complementary Alus were extracted using the pairwise alignment function (type = 'global') of the Biostrings package (V2.40.2), in R. The score criterion was set at > -100.

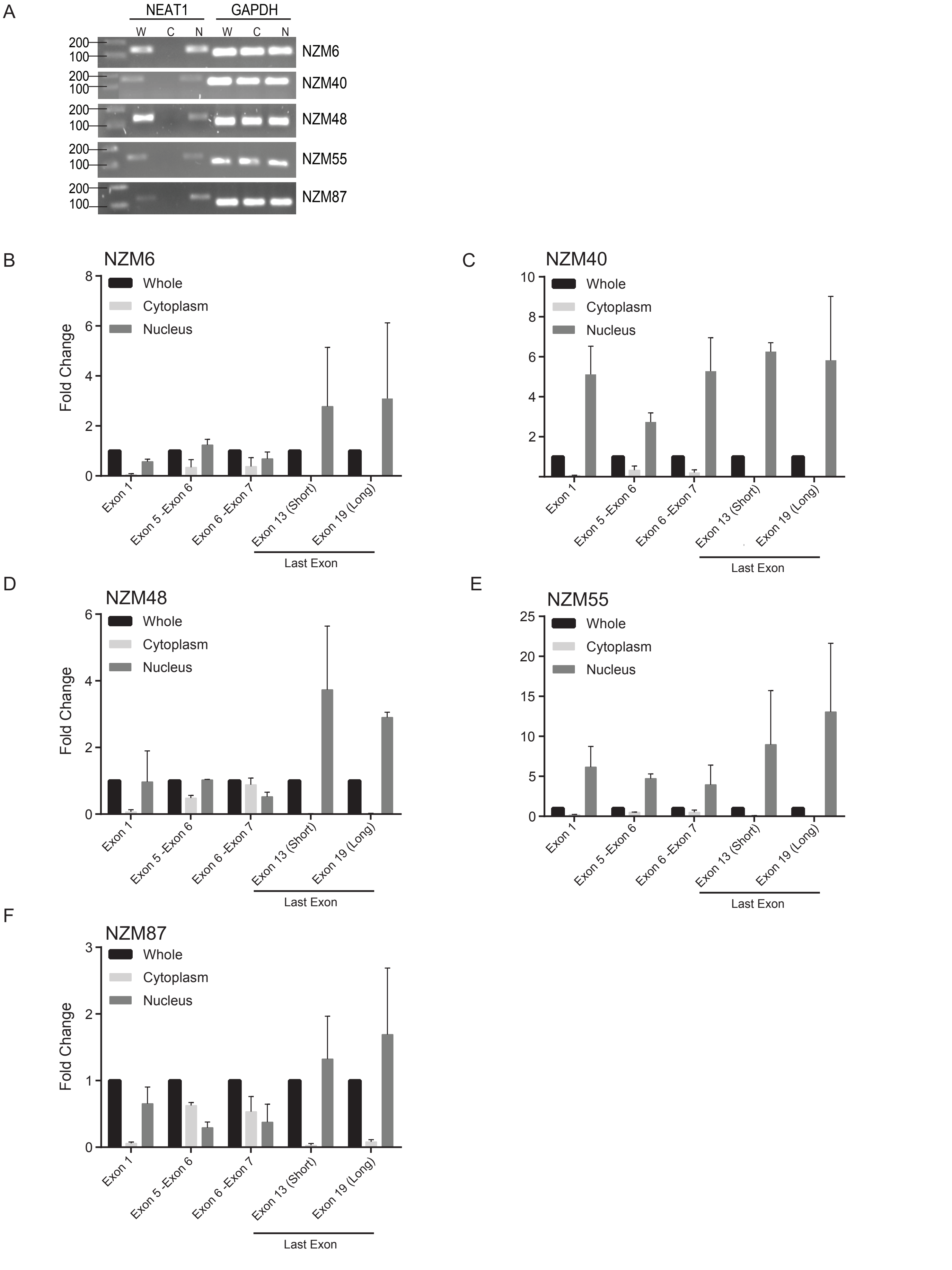

Figure S6. Subcellular localisation of linear ANRIL isoforms in melanoma cell lines. (A) The purity of the cell fractions was checked by PCR using NEAT1 (nuclear marker) and GAPDH in NZM6, NZM40, NZM48, NZM55 and NZM87 cell extracts fractionated into nuclear and cytoplasmic fractions. (B-F) Linear ANRIL exons localised in the nucleus for NZM6 (B), NZM40 (C), NZM48 (D), NZM55 (E) and NZM87 (F) detected using qPCR and the same primer sets as described previously (Fig 2A). The fold change relative to whole cell is shown.

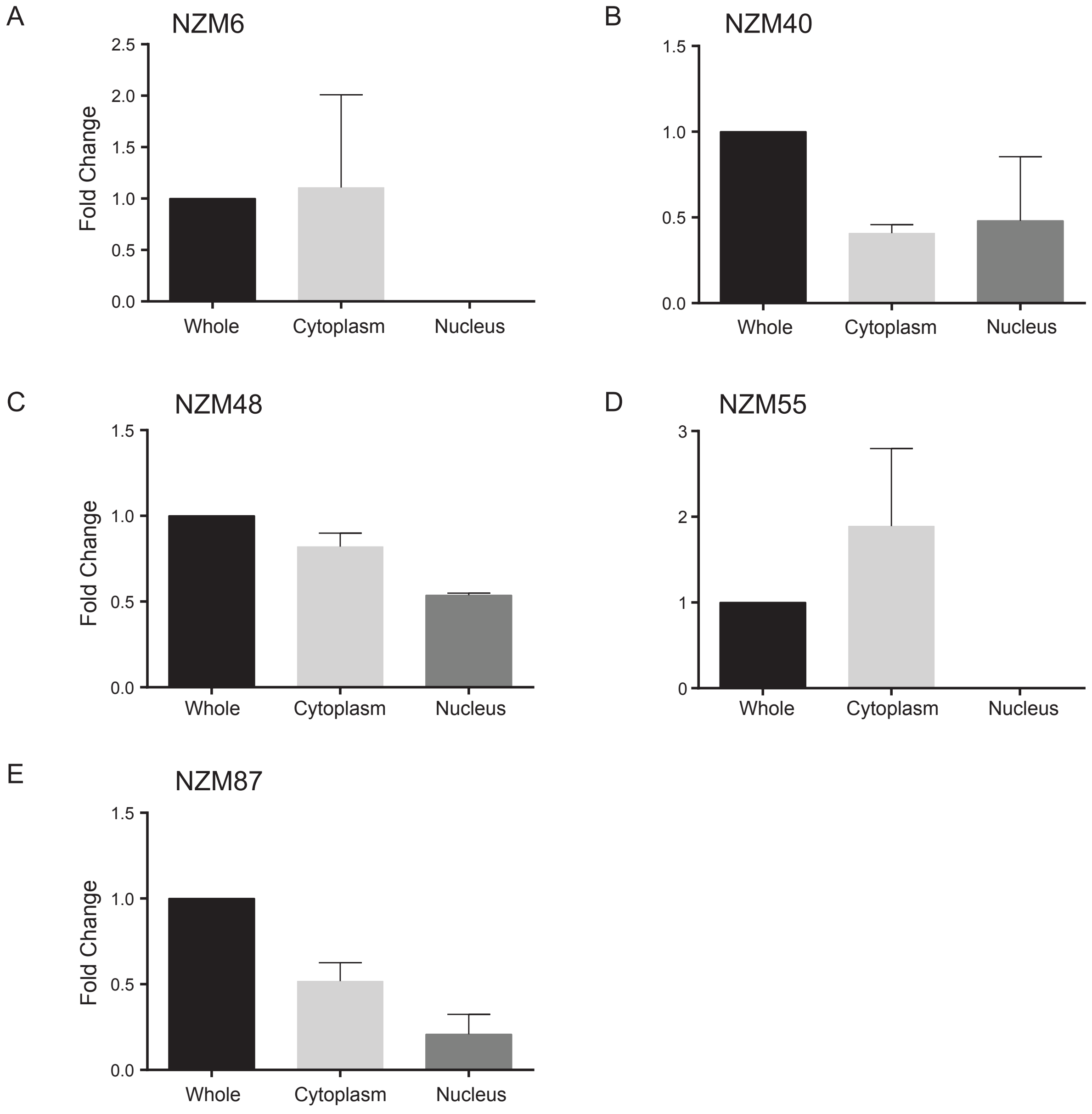

Figure S7. Subcellular localisation of circANRIL in different melanoma cell lines. (A-E) Localisation of circANRIL in fractionated cells using the exon 14 - 5 junction primer set. The fold change relative to whole cells is shown for NZM6 (A), NZM40 (B), NZM48 (C), NZM55 (D) and NZM87 (E) cells.
