# Supplementary material for: Multiple Isoforms of ANRIL in Melanoma Cells: Structural Complexity Suggests Variations in Processing
Source: Int J Mol Sci. 2017 Jun 27;18(7):1378. doi: 10.3390/ijms18071378 (PMC5535871; doi:10.3390/ijms18071378)
Supplement: Supplementary file 1 [file ijms-18-01378-s001.zip › ijms-202411-supplementary tables.pdf]

Table S1. List of primers used.

Primer set	Sequence (5′–3′)	Target
ANRIL		
1F	CCCTGTCGAGGAACAGCTAA	Exon 4 – 5
1R	ATCTGGTGGCCAGAAAACAG	
2F	TACATCCGTCACCTGACACG	Exon 1
2R	CCTCGCTTTCCTTTCTTCCT	
3F	GCCTCATTCTGATTCAACAGC	Exon 5-6
3R	GACTCGGGAAAGGATTCCAG	
4F	AACCGGGGAGATCTATTTGG	Exon 6-7
4R	GGTGTGGTGTCTCACACCTG	
5F	TTTCCTACGAAGCTGGGTGA	Exon 13b (Short isoform)
5R	AAACCCAACAAGATAGAGAAGCA	
6F	TGCTTACCTAGTGCCAGATGC	Exon 19 (Long isoform)
6R	TCAAATCCCAGCCAATTACC	
Circular *ANRIL* exon 14 – exon 5 junction and outward facing primers		
7F	AGTGGCAGGAATTTGGGAA	Exon 14-5
7R	ATCTGGTGGCCAGAAAACA	
Exon 2-F	GTTAGGGTGTGGTATGTGC	Exon 2
Exon 2-R	GAGAGGAGCTGAGGAATCA	
Exon 4-F	GACTACCTGCCTGCCCTGT	Exon 4
Exon 4-R	TATTTAACTTCTCTCTTTCT	
Exon 6-F	AATGTATCTAACTCCAAAG	Exon 6
Exon 6-R	CACACCTAACAGTGATGCT	
Exon 7-F	CCCGGCGGATAGAGAGAAT	Exon 7
Exon 7-R	ACACCTGTAATCCCAGCAC	
Exon 8-F	TGGGAATTCTGGGAGAAAC	Exon 8
Exon 8-R	CCTCCCACCATGATTCTGA	
Exon 14-F	GGGGCCATTCATATGAGAG	Exon 14
Exon 14-R	ATCACTGTGCTCCTCATTCC	
Exon 16-F	TACACACTTGAAGATGGTG	Exon 16
Exon 16-R	CTATTGCATTTGATCTTACG	

Table S2. Back-spliced junction prediction for reverse complementary intron pairs (can possibly go with Figure 5C).

**Reverse complementary intron pairs**	**Predicted back-splicing events**
14-1	14-2
12-1	12-2
11-1	11-2
11-6	11-7
11-7	11-8
6-1	6-2
7-6	7-7
7-5	7-6
5-1	5-2

Table S3. Back-spliced junctions validated in melanoma cells due to possible pairing of inverted Alu repeats in introns flanking either sides of back-spliced exons.

Backsplicing	Intron_1	Intron_2	Alu in intron_1	Alu in intron_2
2-6	1	6	Chr9:22007076-22007388	Chr9:22054786-22055051
2-6	1	6	Chr9:22017143-22017337	Chr9:22054786-22055051
2-6	1	6	Chr9:22017143-22017337	Chr9:22056234-22056365
2-6	1	6	Chr9:22018051-22018262	Chr9:22054786-22055051
2-6	1	6	Chr9:22018374-22018669	Chr9:22054786-22055051
2-6	1	6	Chr9:22023450-22023742	Chr9:22054786-22055051
6-7	5	7	Chr9:22047778-22048093	Chr9:22057525-22057818
